# Can Copper Products and Surfaces Reduce the Spread of Infectious Microorganisms and Hospital-Acquired Infections?

**DOI:** 10.3390/ma14133444

**Published:** 2021-06-22

**Authors:** Joji Abraham, Kim Dowling, Singarayer Florentine

**Affiliations:** 1 School of Engineering, Information Technology and Physical Sciences, Mt Helen Campus, Ballarat, VIC 3353, Australia; k.dowling@federation.edu.au; 2 Department of Geology, University of Johannesburg, Johannesburg 2006, South Africa; 3 Future Regions Research Centre, School of Science, Psychology and Sport, Federation University Australia, Mt Helen Campus, Ballarat, VIC 3353, Australia; S.florentine@federation.edu.au

**Keywords:** coronavirus, COVID-19 pandemic, health care, infection control, microbial infections

## Abstract

Pathogen transfer and infection in the built environment are globally significant events, leading to the spread of disease and an increase in subsequent morbidity and mortality rates. There are numerous strategies followed in healthcare facilities to minimize pathogen transfer, but complete infection control has not, as yet, been achieved. However, based on traditional use in many cultures, the introduction of copper products and surfaces to significantly and positively retard pathogen transmission invites further investigation. For example, many microbes are rendered unviable upon contact exposure to copper or copper alloys, either immediately or within a short time. In addition, many disease-causing bacteria such as *E. coli* O157:H7, hospital superbugs, and several viruses (including SARS-CoV-2) are also susceptible to exposure to copper surfaces. It is thus suggested that replacing common touch surfaces in healthcare facilities, food industries, and public places (including public transport) with copper or alloys of copper may substantially contribute to limiting transmission. Subsequent hospital admissions and mortality rates will consequently be lowered, with a concomitant saving of lives and considerable levels of resources. This consideration is very significant in times of the COVID-19 pandemic and the upcoming epidemics, as it is becoming clear that all forms of possible infection control measures should be practiced in order to protect community well-being and promote healthy outcomes.

## 1. Introduction

History indicates that many ancient civilizations used copper products in their home environment and palaces for ornamental purposes. The ancient Egyptians (2000 BC), the Greeks (400 BC), the Indians, the Aztecs, the Mesoamericans, and Hellenistic civilizations all used copper for sterilising water and for many treatment purposes, even though they were clearly not aware of the microbial world [1,2]. Later, Victor Burq, a well-known 18th century physician in Europe, recognised that copper workers in Paris were apparently immune to the cholera epidemic, and this observation led to the development of metallotherapy cures for several diseases [3]. This trend continued until the remarkable development of antibiotics [3,4]. However, in recent times, where some bacteria have started showing antibiotic resistance, the biomedical community has begun to return to copper and found that the material and its alloys are effective against several microbial pathogens such as *Staphylococcus aureus (S. aureus)* [5], *Bacillus subtilis (B. subtilis)* [6], *Escherichia coli* (*E. coli)* [7], *Legionella pneumophila (L. pneumophila)* [8], and some fungi and viruses [9,10].

It is also noted that copper is an essential trace element required by humans [11], with the recommended daily intake for an adult being 0.9 mg [12]. Many wound-healing medicines were laced with copper due to its antimicrobial property [13], and copper-made intrauterine conceptive devices assisted with birth control measures across the world for many years [14].

It is confirmed that copper is a self-sanitising metal, acting on human pathogens in a way that does not let them survive exposure to copper or copper alloy surfaces for any reasonable length of time. This property is not seen with other common surface materials such as stainless steel, aluminium, and plastic [3,15,16,17], which is a cause for some concern in the current pandemic environment. Regarding the efficacy of copper surfaces, testing in an independent microbiology laboratory has led to 300 various copper surfaces being registered with the United States Environmental Protection Agency (USEPA) in 2008 [3,18]. The registration includes the following statement: “When cleaned regularly, the antimicrobial copper alloy surface kills greater than 99.9% of bacteria within two hours and continues to kill more than 99% of bacteria even after repeated contamination”. This claim acknowledges that copper and its alloys brass and bronze can kill potentially deadly bacteria, and sometime later, it was further understood that copper nanoparticles (Cu-NPs) and laser textured copper also show enhanced antimicrobial activity [19,20].

Despite this health benefit, less costly steel, aluminium, and plastic products have largely supplanted the place of copper in the modern world, although it is noted that some Asian countries continue the use of copper products, specifically as kitchen and dining utensils. As a consequence of these observations, this paper asks whether, during this time of COVID-19 and other upcoming pandemics, the increased use of copper products and surfaces in the home, hospital environments, and public places can reduce the spread of microbial infections.

The objective of this review article is to highlight the biocidal or contact-killing property of the copper metal and its alloys and to investigate the potential use of copper products and the installation of this metal in common touch surfaces in the healthcare and food industries, public places, public transports, and the home and office environments during the current and post-COVID-19 period to reduce the pathogenic microbial spreading. This article is prepared after reviewing several other published literatures collected through significant databases such as Google Scholar, PubMed, Scopus, Medline, and Web of Science using the keywords “antimicrobial property of copper”, “antimicrobial copper”, “copper and microbes”, “antibacterial copper”, “antiviral copper”, “antifungal property of copper”, and “copper and HAIs”.

## 2. The Role of Touched Surfaces in Pathogen Spread

Contamination from common touch surfaces (fomites) plays a significant role in pathogen transmissions, which increase the risk of cross-infections in hospitals and public places [21,22]. It is known that, during illness, patients can shed pathogens in large numbers, which can seriously contaminate built environments and public fomites resulting in the spreading of pathogens between inanimate objects and people, and vice versa [23,24]. The infections that spread through fomites in the hospital environment are called hospital-acquired infections or healthcare-associated infections (HAIs), which have been shown to have initiated morbidity and mortality cases across the globe [25,26]. In the USA, around 4.5% of hospitalised patients develop HAIs, resulting in 1.7 million cases in 2002 [27]. This contributed to about 100,000 mortalities annually, a total that exceeds that of cancer, AIDS, and road accidents combined and accounts for a US$ 35–45 billion additional annual expenditure to the health industry [27]. However, according to the Centres for Disease Control (CDC), the HAIs reduced to 3%, causing 687,000 annual cases and 72,000 deaths in 2015 [28]. Europe and other continents are also not free from HAIs (Table 1). In Europe, over 3.8 million patients are affected by HAIs (both in hospitals and long-term care facilities), causing more than 16 million additional hospital days, 90,000 deaths, and an additional expenditure of US$ 8.3 billion (EUR 7 billion) annually [29,30,31]. It is also assessed that HAI patients required longer hospital stays (21.6 vs. 4.9 days), have higher readmission rates (29.8% vs. 6.2%) and have higher mortality (9.4% vs. 1.8%) compared to non-HAI patients [26,32]. Thus, the position and nature of a fomite have a significant role in the direct or indirect transmission of human pathogens, specifically during epidemics and pandemics.

In addition to these concerns within the health industry, microbial contaminations and spread are also a significant challenge in the food industry. According to the World Health Organisation (WHO), food containing pathogens can generate more than 200 types of diseases, which have caused 550 million people to fall ill and 230,000 to die annually across the world [37]. When it comes to the USA, the CDC estimates that around 48 million people become sick annually from food contamination, causing 128,000 to require hospital treatment and 3000 deaths [38], which causes a US$ 15.5 billion economic burden [39]. Furthermore, the 2018 World Bank report shows that the yearly economic burden of foodborne disease in low and middle-income countries is USD 110 billion, including medical expenses and production loss [40]. Significant food-contaminating bacteria are *Salmonella*, *Campylobacter, E. coli*, *Listeria,* and *Vibrio cholera (V. cholera)* [37]. Unsurprisingly, the WHO considers food safety as a high public health priority [37].

Apart from the health and food industries, several community gathering centres are potential microbial pathogen transfer places. These areas include local, interstate, and international transportation hubs, educational institutions, public and private offices, restaurants, cafes, hotels, the accommodation industry, factories, places of worship, and public gymnasia [24,41]. Over the past two decades in the USA alone, 62 million adults were reported to be infected with the common cold annually, causing 25 million doctor visits, 20 million days of absence from work, and 22 million days of absence from schools [42]. This rate means one adult per second will contract a common cold virus, mostly arising from contact with the built environment. In a similar but more concerning instance, the Sri Petaling gathering in Malaysia at the end of February 2020 was responsible for the transmission of hundreds of COVID-19 cases and is now considered to be linked to 35% of the total COVID-19 cases in Malaysia during the first wave [43]. It has also been reported that various religious congregations fuelled the COVID-19 spreading in India, Northern Italy, South Korea, and Iran [43,44]. It is likely that, apart from the aerosol transmission, some of the COVID-19 cases might have been through the built environment [45]. We cannot rule out the possibility of fomite spreading since it is observed that SARS-CoV-2 can be active up to 28 days on common indoor surfaces) [46], and 10^8^ viral copies can be present in 1 mL of the sputum [45].

Within the healthcare environment, the presence of methicillin-resistant *staphylococcus aureus* (MRSA) and vancomycin-resistant enterococci (VRE) are emerging as high-profile challenges, but, by comparison, more concern really relates to the arrival of antibiotic-resistant Gram-negative bacteria such as *E. coli*, *Klebsiella pneumonia (K. pneumonia)*, *Pseudomonas aeruginosa (P. aeruginosa)* and the *Acinetobacter* species [47]. This poses a real and distinct challenge to the medical world. Here might be the role for copper and copper alloy products, since surfaces can be equipped with this material, thus controlling and reducing the bioburden in the health and food industries and public places.

## 3. Establishing the Case for the Re-emergence of Copper Surfaces

Recent investigations have determined that metallic copper has a particularly effective antimicrobial property known as contact-killing. Therefore, it is appropriate to present a brief look at the wider issue of general metal activity [3]. Majno [48] experimentally demonstrated the antimicrobial property of copper, showing that no wound bacteria will grow in the proximity of copper. Other researchers have observed that there is less contamination with *Legionella* in water pipes made of copper than those of steel and plastic [49]. In addition, copper-impregnated wound dressings were tested in an independent laboratory in the UK and revealed that the reduction of several bacteria strains, including MRSA, compared well to the traditional silver (Ag) adhesive dressings [50].

It is now well established that, whilst stainless steel is the most widely used metal in health and food industries and public places due to its corrosion resistance and clean appearance, it does not have any inherent antimicrobial properties [3,15,17,27]. Indeed, studies have shown that microbes, including SARS-CoV-2, can stay viable on stainless steel for three to 28 days, whereas copper inactivates the same virus within four hours [46,51]. Several other laboratory studies have indicated that human pathogens can only survive on copper or copper alloy surfaces for a maximum of several hours at room temperature, with many dying within minutes [3,27,31]. Many researchers have examined the contact-killing property of copper in healthcare environments and have observed that copper hospital beds have 95% fewer bacteria than normal beds [17,27,52]. It is also observed that the installation of copper alloys on the high-touch surfaces in an athletics centre has lowered bacteria counts by 94% [53].

It is sobering to recall that MRSA can exist on hospital surfaces as long as 360 days [54], and *Clostridium difficile (C. difficile)* can survive for several months [16]. Among the two, MRSA is of particular concern due to the increasing number of infections [27,52]. Against this backdrop, we note that recent studies have found that copper and its alloys can kill MRSA, *Acinetobacter* strains, *E. coli*, several other bacteria strains, and can inactivate norovirus, coronavirus (SARS-CoV and SARS-CoV-2), and many other viruses [3,51,55,56,57,58]. Of considerable relevance here is that it has been shown that not only pure copper surfaces but copper alloys such as bronze and brass have also been shown to kill or inactivate potentially deadly microbes [59,60,61].

They also offer long-term protection [18], suggesting that copper has a significant role as a control barrier against spreading harmful pathogens and can be used to supplement standard infection control practices [62]. This is an important issue since Barker et al. [63] observed that a contaminated finger could sequentially transfer a virus to up to seven previously clean surfaces.

## 4. A Survey of the Antipathogenic Properties of Copper and Its Alloys

### 4.1. Copper as an Antibacterial Agent

The contact-killing ability of copper surfaces was studied with respect to bacteria in the early 1980s due to the emerging of hospital superbugs. In 1983, Kuhn [64] compared the bioburden on doorknobs made of brass and stainless steel and found that brass doorknobs showed reduced pathogenic growth in the healthcare environment compared to the stainless steel variety. Recently, Schmidt et al. [17] replaced the normal plastic rails of hospital patients’ beds with copper and then tested for the microbial burden. It was found that the plastic surfaces on the control beds exceeded the recommended amount of bacterial concentrations, but it was not so on the copper beds (94% lower). Salgado et al. [32] installed copper alloys on common touch surfaces in the hospital environment such as bed rails, overbed tables, intravenous poles, arms of visitor’s chairs, nurses’ call buttons, computer mouses, the bezel of the touch screen monitor, and palm rest of laptop computers in three intensive care units (ICU) of three large hospitals in the USA. A total of 650 randomly selected patients were observed in 16 ICU rooms, with eight copper alloy fitted and eight control rooms. The results showed that the MRSA and VRE concentrations were significantly lower (0.071 vs. 0.123; *p* = 0.020) in the copper-alloy equipped ICUs compared to the standard ICUs [31]. It was also noticed that placing copper alloy surfaces in the ICU rooms reduced the risk of HAI by more than half during the study period, and no HAI outbreak of epidemiologically important organisms occurred in copper-alloy equipped ICUs. A detailed study was conducted in another 16 ICU rooms (eight experimental rooms and eight control rooms) of three hospitals in the USA over 21 months, replacing the normal hand-touch steel surfaces with copper, and this study also found that copper materials at the hand-touch surfaces significantly reduced the microbial burden (698 vs. 6102 CFU per 100 cm^2^, 88% reduction) [57].

**Figure 1:**
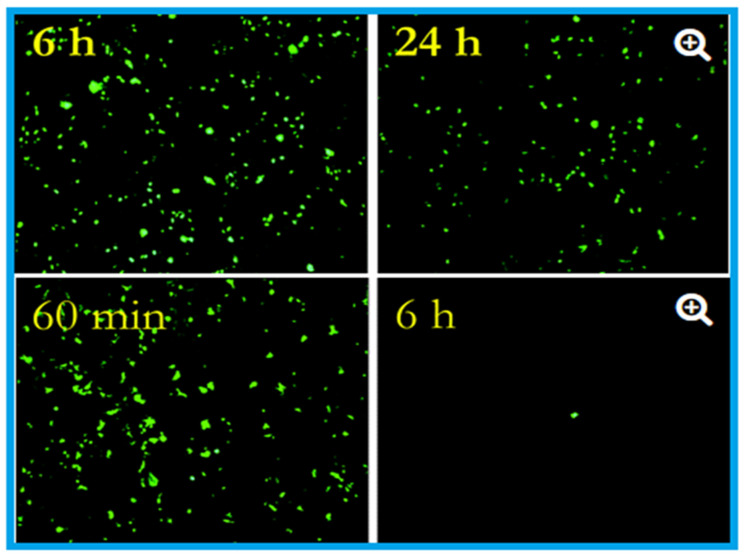
Effects of influenza A virus on steel surface (top) and copper surface (bottom). The influenza virus was cultured in R-mix vials that contain a monolayer of mink lung and human laryngeal carcinoma cells grown on glass coverslips. This was inoculated into sterile coupons of copper (C11000) and steel (20 µL virus suspension with 10^8^ virus particles per millilitre) for the experimental purpose and kept at room temperature (22 ± 2 °C) with a relative humidity of 50 to 60%. Here in the epifluorescent image, the number of green fluorescing cells are proportional to the viral inoculum. After six hours, 10^6^ virus particles were found to be remained viable on the steel surface, and after 24 h, 5 × 10^5^ particles were present, capable of causing cell infection (top). In contrast to the steel surface, on copper, the virus particles reduce to 5 × 10^5^ after 60 min (the equivalent of 24 h of exposure on stainless steel), which reduced to 5 × 10^2^ after six hours (nearly 4 log reduction). After 24 h of incubation, 500,000 virus particles were present on stainless steel, but 500 only seen after six hours on the copper surface. Adapted with permission from Ref. [92]. Copyright 2007, American Society for Microbiology.

A three-year-long study was conducted in France in five extended care facilities, replacing the doorknobs and handrails with copper alloys. Around 1400 samples were collected and analysed and found that copper doorknobs and handrails revealed significantly less microbial burden (59% and 33% reduction, respectively) than the normal doorknobs and handrails [65]. Other studies conducted in the health care environment have also reported the benefits of replacing plastic hospital beds with copper or copper alloys due to the significant reduction in the microbial burden [66,67,68,69]. However, the studies revealed that the contact-killing property increases with an increase in copper concentration, and a minimum of 60% copper concentration is required in alloys to get the best result [16,57,70,71,72]. Souli et al. [72] studied the antibacterial efficacy of two copper coatings (99% and 63% copper concentrations) on various multi-drug resistant Gram-negative pathogens responsible for nosocomial infections such as *E. coli*, *Enterobacter* spp., *K. pneumonia*, *P. aeruginosa,* and *Acinetobacter baumannii (A. baumannii).* They found that copper coatings worked against all strains of the above microbes, with those having greater than 99% copper concentration being able to kill the microbes below six hours (2 h for *A. baumannii*, 3 h for *Enterobacter* spp., 5 h for *K*. *pneumoniae*, and 6 hr for *P*. *aeruginosa*) [72]. The contact-killing efficacy of copper surfaces on *Clostridium difficile (C. difficile)* (a major cause of hospital-acquired infection globally) showed similar characteristics to alloys with higher copper concentrations (>70% copper), killing the *C. difficile* (vegetative cells and spores) after 24–48 h [73]. This microbial contact-killing efficacy of copper has also been found to be successfully applied in wound dressings (Figures 1–3 in [50]).

Apart from the copper concentration, the biocidal efficacy of a surface depends on many other factors such as atmospheric temperature, humidity, length of exposure, microbial type, and concentration [50]. It does seem that contact-killing capability remains high across all standard temperature ranges [74,75]. Noyce et al. [76] studied the characteristics of copper alloys at 22 °C and 4 °C with MRSA and found that at 22 °C, all the three MRSA strains (10^7^ MRSA, EMRSA-1, and EMRSA-16) were completely killed after 45, 60, and 90 min respectively, but it took six hours to completely eradicate these strains at 4 °C [76]. Michels et al. [75] observed a >6.4 log reduction of MRSA when the temperature was 35 °C and humidity was >90%, whereas it was a >6.1 log reduction when the temperature was reduced to 20 °C. It is also noted that the alloys with higher copper concentration (85% and above) were able to completely kill *E. coli* bacteria at a lower temperature [77]. Similar studies were conducted by Wilks et al. [78,79], who found that antibacterial properties exist at all temperatures but were superior when copper concentrations exceeded 85%. Testing for MRSA at 20 °C on four copper alloys—C19700 (99% Cu), C24000 (80% Cu and 20% Zn), C22000 (90% Cu and 10% Zn), and C77000 (55% Cu, 27% Sn, and 18% Ni)—showed that for C19700, there was a drop off within 75 min and for C22000, drop off was after 270 min. Both are considered to be more than 99% effective [75]. In a similar investigation, Bleichert et al. [80] looked at the biocidal effects of copper surfaces on bacterial and viral biothreat agents and revealed that cells of bacterial biothreat agents exposed to copper surfaces are inactivated within a few minutes. On the other hand, the cells on the control surface (stainless steel) showed a slower decline of the viable cells over time [80].

Whilst most recent studies were in the hospital environment, Inkinen et al. [81] decided to study the antibacterial efficacy of copper in different environmental settings such as retirement homes, kindergartens, and office buildings. Copper replaced traditional materials at the common touch surfaces (such as door handles, light switches, corridor handrails, closet touch surfaces, toilet flush buttons, floor drain lids, and toilet support rails). The study found that the copper surfaces had a lower bacterial load than the reference products and concluded that copper touch surfaces functioned efficiently as an antibacterial surface [81]. It was found that *C. difficile* can form spores and survive on dry surfaces for up to five months, and cannot be killed by hospital-grade disinfectants [27]. However, copper, including its alloys with greater than 70% copper, can kill the *C. difficile*, including the spores [27]. The antimicrobial property of copper regarding *C. difficile* was also studied by Wheeldon et al. [82] in a clinical setting using carrier test methods against dormant and germinating spores and vegetative cells for three hours in the presence and absence of organic matter. It was found that within 30 min, the copper surface destroyed the vegetative cells and reduced the viability of spores exposed to germination within an hour, giving an additional positive signal for using copper in the hospital environment to reduce infection. Besides copper metal and alloys, a copper coating on a steel surface was also found to enhance the antibacterial property of the steel [83].

In the food industry, most bacterial contamination is due to *E. coli* O157 and is responsible for large-scale food recalls [77]. Noyce et al. [77] studied the efficacy of seven cast copper alloys with copper concentration ranges from 61% to 95% to investigate the ability to reduce *E. coli* strains in the food industry environment. The study found that without the addition of beef extract, three alloys completely killed the *E. coli* inoculum within six hours of exposure at 22 °C, but at a lower temperature (4 °C), only the copper alloys with higher copper concentration (>85%) were able to significantly reduce the inoculum [77].

*Listeria monocytogenes (L. monocytogenes),* commonly found in soil, water, plant materials, and animals (including humans), are of considerable concern in the food industry [79]. It has been recognised as a human pathogen since 1929, and records show that *Listeria* infections affect around 2500 people every year in the US, causing 500 deaths annually [79]. It can be critical to pregnant women, the elderly, and immunocompromised people [84]. The bacteria cause Listeriosis, whose symptoms are often septicaemia, encephalitis, spinal meningitis, and corneal ulcers, including pneumonia, which is considered the cause of miscarriage and even death [85,86]. Aisha [87] investigated copper alloys’ antimicrobial effect in killing *Listeria* and found that copper ions are very effective. Wilks et al. [79] also studied copper’s efficacy in killing *Listeria*, and found no viable *Listeria* on any copper alloys after 60 min (5 Log reduction), whereas viable cells were found on stainless steel even after 24 h. Furthermore, they reported that a new alloy called New Silver (65% Cu, 18% Ni, and 17% Zn) also inactivated all bacteria within 90 min of exposure. All these studies support the conclusion that copper products and surfaces can be effectively used in many locations, especially in the health industries and public places, to reduce the bacterial burden and subsequent diseases.

Even though many studies mentioned the influence of copper surfaces in reducing the microbial burden, a review by Cochrane conducted in Australia mentioned that there is only limited evidence available to support the use of environmental fittings with antimicrobial properties in preventing infections with multi-resistant bacterial organisms [88].

### 4.2. Copper as an Antiviral Agent

The antiviral activity of copper was studied as early as 1958 by Bauer [89], whose work was followed by many researchers who demonstrated the efficacy of copper against many viral strains [1,10,15,51]. Published studies (Table 2 and Table 3) confirmed the contact-killing property of copper surfaces against viruses such as influenza virus, norovirus, monkeypox, vaccinia virus, human immunodeficiency virus (HIV), SARS-CoV, and SARS-CoV-2 [3,10,51,89,90]. Researchers at the University of Southampton showed that they could significantly prevent the spreading of influenza using copper surfaces, and it was further revealed that the influenza virus could be eradicated within six hours of exposure to a copper surface [91]. These researchers placed two million active units of influenza A (H1N1) virus on a sheet of copper (C1100, which is pure copper under ISO standards) and stainless steel (S30400) (Figure 1). After 24 h, the virus on the steel had declined to 500,000 units, but only 500 viruses were found to be active after six hours on the copper [62,91,92].

**Figure 2:**
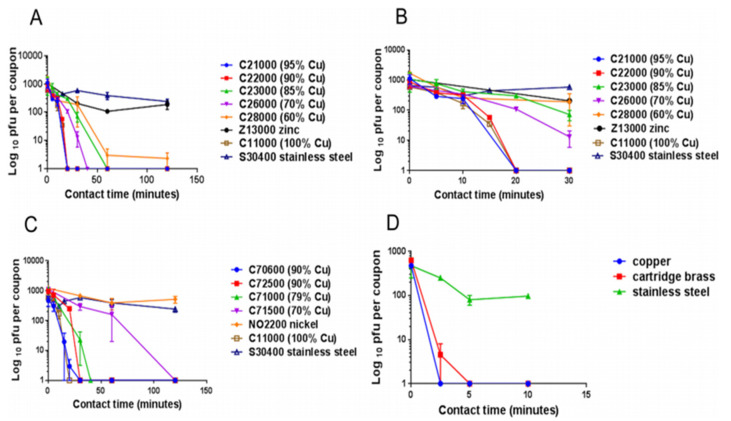
The panels describe the rapid contact-killing efficiency of various copper alloys (varies from 60% copper concentration to 100%) on human coronavirus-229E (HuCoV-229E), which causes the common cold. Initially, around 10^3^ PFU HuCoV-229E (20 µL infected cell lysate) was applied on one sq. cm copper alloy coupon(s) with various copper concentrations, including stainless steel, nickel, and zinc (as control metals). (**A**) It was found that the coronavirus was inactivated less than 40 min on brass coupons and less than 120 min on copper-nickel alloy (containing less than 70% copper). Surprisingly the alloy with 70% copper showed quick antiviral activity than the alloy with 85% copper. (**B**) The observation showed an initial time lag on all alloys and metals, followed by rapid inactivation on copper coupons. The control metals stainless steel and nickel did not show any anti-coronavirus activity, except zinc, which showed little (significant only after 60 min, *p* = 0.046). (**C**) When the copper concentration was reduced to 70%, it took approximately 80 min more to inactivate all the viruses compared to one with 79% copper. (**D**) When the same inoculum was applied at 1 µL per sq. cm, the coupons inactivated the virus eight-time faster. The experiment showed that the concentration of copper and the amount of virus are significant factors in showing the antiviral activity. Adapted from Ref. [93].

Warnes et al. [93] tested the capability of inactivating one corona group virus, (229E), that can cause common colds and pneumonia. They found that the virus became inactivated immediately after being kept on copper, but it stayed viable for five days on stainless steel and glass (Figure 2). Similar to bacteria, inoculation efficiency for the virus also depends on temperature, humidity, copper concentration [3,76,77,78,93,94,95], length of exposure, and microbial density [76,78,79,96].

Norovirus is highly infectious, causing viral gastroenteritis, and is spread through touch surfaces [97]. Warnes and Keevil [90] investigated the antiviral property of copper alloy surfaces against norovirus and found its effectiveness is proportional to the copper concentration in the alloy. Furthermore, they observed that antiviral effectiveness was not very rapid on brass but was very effective on the copper-nickel alloy. It is also found that copper-based filters inactivate HIV-1, which can significantly reduce HIV-1 infection through breastfeeding and blood donation [98]. In addition to this, Noyce et al. [92] indicated that copper surfaces act as a barrier against the avian flu epidemic. Their experiments have shown that, after six hours of exposure on a copper surface, 99.9% of the two million active H5N1 virus particles involved in the experiment became inactive.

**Table 2:** Biocidal activity of copper on various viruses.

Types of Virus(es)	Effect(s)	Reference(s)
SARS-CoV-2	The virus was active only up to 4 h on the copper surface	[51]
SARS-CoV	The virus was active only up to 8 h on the copper surface	[51]
Influenza A virus	After incubation for six hours on copper 99% of the viral particles were inactivated	[92]
Influenza A virus	Solid-state copper oxide (Cu_2_O) inactivated the influenza A virus	[99]
Human coronavirus HuCoV-229E	Active only 20 min on copper surface	[93]
Hepatitis C virus (HCV)	Copper oxide-NPs significantly inhibit the infectivity of HCV, both at the entry and attachment stages	[100]
Murine norovirus-1 (MNV-1)	Copper alloy (65 to 99.9% Cu) dry surfaces inactivated the MNV-1	[90]
Vesicular Stomatitis Virus Coxsackie Virus-B4 Respiratory Syncytial Virus	Curcumin-copper synthesised compound found to effective against these viruses and could be utilised for the development of vaginal microbicidal gel	[101]
Feline Calicivirus (FCV)	CuI-NPs reduced the infectivity of FCV by order of seven magnitude	[102]
H1N1 Influenza Virus 2009 Pandemic	CuI-NPs showed antiviral activity against influenza A virus of swine-origin	[10]
Human Immunodeficiency Virus-1 (HIV-1)	When exposed to copper oxide, the HIV-1 infectivity inhibited in a dose-dependant manner	[98]
Polio Virus	Copper sulphate (20 mg/L) completely inactivated the polio virus in the presence of hydrogen peroxide	[103]
Herpes Simplex Virus (HSV)	Reducing agents such as ascorbic acid, hydrogen peroxide and cysteine enhanced the antiviral property of copper	[104]

### 4.3. Copper as an Antifungal Agent

The antifungal property of copper was first identified in 1761 when it was found that grain seeds soaked in copper sulphate solutions could inhibit the seed-borne fungi, but it took more than 100 years for the more sophisticated development of the fungicide “Bordeaux mixture” (developed by Pierre-Marie-Alexis Millardet and used in the USA) and “Burgundy mixture” (used in France) [105]. Copper sulphate and lime mixtures were sprayed onto grape wines to make them mildew-free, prevent fungal infection in other plants, and control the algal growth in water reservoirs as well on timber, and were also found useful in preserving fabric [105]. This experience again shows that even though the antimicrobial property of copper has been used in the agriculture sector for controlling fungal and bacterial infections for many years [106,107], it has come to the healthcare environment very lately.

A peer-reviewed study of the fungicidal property of copper was carried out in the 1950s, finding that copper, including copper compounds, are effective in killing several fungi and yeast, including *Candida albicans (C. albicans)* [108,109], *Aspergillus niger (A. niger)* [107], and *Aspergillus carbonarious (A. carbonarious)* [110]. Indeed, many thousand tons of copper-based antifungal agents, specifically copper sulphate and copper hydroxide, are annually used across the globe for agricultural purposes [111]. It is also used in wood processing to prevent roof moss formation and as an algae-resistant roofing system in the 3M industry. The biocidal efficacy of copper against *Aspergillus* and *Fusarium* species as well as *Penicillium chrysogenum (P. chrysogenum)* and *C. albicans* was studied by Weaver et al. [9], who found that copper surfaces were able to kill most of these fungi and were able to prevent germination of new spores. The mechanism for control with bacteria and fungi is similar as inoculation starts with membrane damage, followed by enlargement and disappearance of vacuoles and the onset of oxidative stress. *Candida* spp. can commonly survive in the healthcare environment and can cause HAIs [112]. The efficacy of copper-sputtered polyester surfaces (Cu-PES) was tested against azole-resistant *C. albicans* and *Candida glabrata (C. glabrata)* under dark and low-intensity visible light, with the results showing that under low-intensity visible light, the Cu-PES exhibited fungicidal activity against both strains within 30 min of exposure [112]. Of interest, it was found that, in addition to the pure copper surfaces, many copper compounds, such as the copper (II) complex of quinoline-2, could act as antifungal agents [113]. Ghasemian et al. [114] tested the antifungal efficiency of Cu-NPs against filamentous fungi (*Alternaria alternate (A. alternate), Aspergillus flavus (A. flavus), Fusarium solani (F. solani),* and *P. chrysogenum)* and found that Cu-NPs are very effective control agents, finding that particle size is a significant factor in antimicrobial activity. Two other studies also found that Cu-NPs are effective against *Candida* species [115,116]. The contact-killing ability of copper for various microbes, including fungi, is summarised in Table 3.

**Table 3:** Details of contact-killing or inactivation of microbes by copper surfaces. Adapted with permission from Ref. [3]. Copyright 2007, American Society for Microbiology.

Species	Application Method (Wet (W)/Dry (D))	Time to No Viable Forms Detected	Reference(s)
SARS-CoV-2	D, 10^5.25^ 50% (TCID_50_) per mm	4 h	[51]
SARS-CoV	D, 10^6.75–7.00^ TCID_50_/mm	8 h	[51]
Human coronavirus—HCoV-229E	W, 10^3^ PFU	20 min	[93]
Influenza A virus (H1N1)	W, 5 × 105 viruses ^h^	5 h	[92]
*Penicillium crysogenum*	W, (2–300) × 10^5^ spores ^c^	24 h	[9]
*Fusarium solani*	W, (2–300) × 10^5^ spores ^c^	24 h	[9]
*Fusarium oxysporum*	W, (2–300) × 10^5^ spores ^c^	24 h	[9]
*Fusarium culmonium*	W, (2–300) × 10^5^ spores ^c^	24 h	[9]
*Aspergillus niger*	W, (2–300) × 10^5^ spores ^c^	>576 h	[9]
*Aspergillus fumigatus*	W, (2–300) × 10^5^ spores ^c^	>120 h	[9]
*Aspergillus flavus*	W, (2–300) × 10^5^ spores ^c^	120 h	[9]
*Candida albicans*	W, 10^5^ CFU ^f^	1 h	[59]
*Saccharomyces cerevisiae*	D, 10^6^ CFU ^k^	30 s	[117]
*Candida albicans*	D, 10^6^ CFU ^k^	5 min	[117]
*Candida albicans*	W, (2–300) × 10^5^ spores ^c^	24 h	[118]
*MRSA ^d^*	W, 10^7^ CFU ^f^	3 h	[59]
*MRSA NCTC 10442*	W, 2 × 10^7^ CFU	75 min	[75]
*EMRSA-16 ^e^ (NCTC13143)*	W, (1–1.9) × 10^5^ CFU ^c^	90 min	[74]
*EMRSA-1 ^e^ (NCTC11939)*	W, (1–1.9) × 10^7^ CFU ^c^	1 h	[74]
*MRSA ^d^ (NCTC10442)*	W, (1–1.9) × 10^7^ CFU ^c^	45 min	[74]
*Acinetobacter baumannii*	W, 10^7^ CFU ^f^	3 h	[59]
*Pseudomonas aeruginosa*	W, 10^7^ CFU ^f^	3 h	[59]
*Klebsiella pneumoniae*	W, 10^7^ CFU ^f^	1 h	[59]
*Mycobacterium tuberculosis*	W, 2.5 × 10^7^ CFU ^f^	5–15 days	[59]
*C. difficile (ATCC 9689) vc&spores*	W, 2.2 × 10^5^ CFU ^c^	24–48 h	[73]
*Pseudomonas aeruginosa PAO1*	W, 2.2 × 10^7^ CFU ^j^	2 h	[74]
*Escherichia coli O157*	W, 2.7 × 10^7^ CFU ^c^	75 min	[75]
*Listeria monocytogenes Scott A*	W, 10^7^ CFU ^c^	1 h	[77]
*Escherichia coli O157*	W, (3–4) × 10^7^ CFU ^c^	65 min	[78]
*Brucella melitensis NCTC 10094*	D, 10^6^ CFU ^k^	<5 min	[80]
*Burkholderia mallei NCTC 3709*	D, 10^6^ CFU ^k^	<5 min	[80]
*Burkholderia pseudomallei NCTC 0816-03*	D, 10^6^ CFU ^k^	<5 min	[80]
*Francisella tularensis FSC 237*	D, 10^6^ CFU ^k^	<5 min	[80]
*Yersinia pestis NCTC 2028*	D, 10^6^ CFU ^k^	<5 min	[80]
*C. difficile germinating spores*	W, 8 × 10^6^ CFU ^i^	3 h	[82]
*C. difficile dormant spores*	W, 8 × 10^6^ CFU ^i^	ua-3 h	[82]
*C. difficile NCTC11204/R20291 vc*	W, (1–5) × 10^6^ CFU ^i^	30 min	[82]
*Different Enterococcus* spp.	W, 10^6^ CFU ^f^	1 h	[96]
*Enterococcus hirae ATCC 9790*	W, 10^7^ CFU ^c^	90 min	[96]
*Escherichia coli W3110*	D, 10^9^ CFU ^k^	1 min	[119]
*Brachybacterium conglomeratum DSM10241*	D, 10^9^ CFU ^k^	A few min	[119]
*Staphylococcus warneri DSM 20316*	D, 10^9^ CFU ^k^	A few min	[119]
*Pseudomonas oleovorans DSM1045*	D, 10^9^ CFU ^k^	1 min	[119]
*Pantoea stewartii DSM30176*	D, 10^9^ CFU ^k^	1 min	[119]
*Acinetobacter johnsoni SM6963*	D, 10^9^ CFU ^k^	1 min	[119]
*Campylobacter jejuni*	W, 4.5 × 10^6^ CFU ^b^	8 h	[120]
*Salmonella enterica*	W, 4.5 × 10^6^ CFU ^b^	4 h	[120]

NB: ua—unaffected; vc—vegetative cells; TCID—tissue culture infectious dose. b—Inoculation with 1.5 mL of culture (4.5 × 10^6^ CFU), kept under humid conditions. c—Inoculation with a 20 µL drop of culture. d—Methicillin-resistant *Staphylococcus aureus.* e—Epidemic methicillin-resistant *Staphylococcus aureus.* f—20 µL of culture spread on coupons. h—inoculation with 20 µL of virion suspension; i—100 µL of dilute culture; j—25 µL of culture spread on coupons with a glass spreader. k—Thin-film applied with a cotton swab.

## 5. Application of Copper Nanoparticles (Cu-NPs)

Nanotechnology is attracting global attention due to its enormous potential in a wide range of applications, and Cu-NPs have attracted more significance both in the health and food industries because of their antimicrobial characteristics. Various methods are used for the preparation of Cu-NPs, such as microwave irradiation, thermal reduction, vacuum vapour deposition, chemical reduction, laser ablation, and polyol [120]. Remyadevi et al. [121] synthesized Cu-NPs using the modified polyol method and carried out the antimicrobial activity against several bacteria (*S. aureus, E. coli, K. pneumonia,* and *P. aeruginosa*) and fungi (*C. albicans, A. niger,* and *A. flavus*). After careful study, they revealed that Cu-NPs exhibit antimicrobial activity, which is strong in bacteria than the fungi [121]. Apart from this, it is noted that Cu-NPs showed antimicrobial property against MRSA, *E. coli*, *Bacillus subtilis*, *P*. *aeruginosa, Salmonella enterica* serotype Choleraesuis (S. Choleraesuis) [115,122], and hepatitis C [100]. After a detailed study and data analysis, Raffi et al. [123] concluded that Cu-NPs with a large surface-to-volume ratio efficiently inactivate *E. coli* bacteria.

The Cu-NPs can be immobilized and coated onto various surfaces to generate or improve antimicrobial activity. In this respect, Cu-NPs- and nanoparticle-impregnated materials, including cloths and plastic, have been shown to exhibit antimicrobial properties, which can be used in various fields, specifically in the health industry [124]. For example, some researchers found that Cu-NP-impregnated face masks showed biocidal activity against human and avian influenza A virus [98].

As mentioned above, the antimicrobial properties of Cu-NP fabrics have been incorporated in textile technology to develop materials suited for use in the health industry, and a specific platform technology has been developed to introduce copper into cotton fibres, latex, and other polymeric materials. The copper oxide NPs (CuO-NPs) (3%–10%) (prepared by a wet chemical method) were microencapsulated by ionic gelation and applied to plain weave cotton fabric, following a pad-dry-cure technique, and it was found that the fabric demonstrated a high level of antimicrobial activity, which could be used in the healthcare environment to reduce the bioburden [125]. In this respect, the studies of Niiyama et al. [19] and Palza et al. [126] are very encouraging. Niiyama et al. [19] determined whether a copper film bedsheet would reduce MRSA infection in a dermatology ward and found that the MRSA count on the sheet coated with Cu-NPs was significantly lower (20–30 colony-forming units, or CFUs) when compared to the non-copper coated bed sheet (6600–11,000 CFUs). Palza et al. [126] tested the antimicrobial behaviour of materials with copper-based additives (copper NPs on plastic matrices) in a hospital environment. As a part of this study, the researchers replaced the normal plastic waiting room chairs with Cu-NPs-embedded chairs, and IV poles made of metals were coated with organic paint impregnated with nanostructured zeolite/copper. They continued sampling once a week for ten weeks and analysed the levels of viable microorganisms. It was found that the copper substrates reduced 73% of the viable microbes in the waiting room chairs and found only low levels of microbes remaining in the IV poles. Apart from this, Harikumar and Aravind [125] investigated the antimicrobial characteristics of Cu-NPs and Cu-nanocomposites against *E. coli* using the well diffusion method and found that the antimicrobial activity increases with an increase in particle dose and contact time.

Research indicates that the antibacterial activity of copper-based NPs is far superior to that of a normal copper surface, mainly because of the small size (high surface area compared to the volume) and higher cell penetration [127]. Several studies indicated that both Cu-NPs and copper oxide (Cu_2_O) induced DNA degradation occurs in Gram-positive and negative bacteria even though the concentration of released ions was far below the normal level for inhibiting bacterial growth [127,128]. This highlighted that with Cu-NPs, the concentration of released ions is less significant than the effect of NP size [128]. Other studies support this finding and have suggested that the size of the Cu-NPs is the major contributing factor for its antimicrobial activity [115,129]. To support this, Padil and Cernik [130] found that small (4.8 +/−1.6 nm) CuO-NPs have significantly higher antimicrobial activity than the larger particles (7.8 +/−2.3 nm), whilst Applerot et al. [127] surmised that the advantage of small particles would be their higher penetration capacity into the microbial covering. In addition to their antibacterial activity, normal copper and Cu-NPs were shown to have antifungal property against several fungi, including C. *albicans* and *Saccharomyces cerevisiae* (*S. cerevisiae*) [114,117], and found that the control mechanism in fungi was similar to that in the case of bacteria [117].

Currently, Cu-NPs are used in various industries and production sectors, but this increased use comes with a cost. Copper NPs have several adverse effects regarding environmental health since, in many regions, these copper NPs have been released into the environment. This is of special concern in the aqueous environment, where it generates health risks to aqueous organisms. It has been observed that copper NPs are not only toxic to bacteria but act on some species of fish and also on mice [131,132,133]. In general, copper, which is released from various industries, normally emerges in two forms, either as dissolved copper or particulate-bound copper, and the toxicity and the mobility of the copper depend on the form [134].

## 6. The Various Applications of Copper in the Built Environment

Cooling towers and potable water distribution systems have recently been determined as the source of hospital outbreaks of several Legionnaires’ diseases [135,136]. However, after several studies, it has been confirmed that the use of a copper-silver ionisation system is the most successful long-term water disinfection system that can be used in the hospital environment [137,138,139,140,141]. In similar investigations, it has been established that in the dental industry, dental cement having copper shows potential antimicrobial properties [142], and in the food industry environment, shifting to copper surfaces and copper-made food carrying, and transportation surfaces produce a significant reduction in foodborne diseases [77,78,120,121,132,143,144]. In addition, thin-film copper oxide (CuO)-coated glass [145], CuO-impregnated degradable phosphate glass fibres [146], and copper alloys also have shown potential biocidal properties against string bacteria spores [73,145]. Drinking glasses made of copper or copper-impregnated glasses were found to reduce the biofilm formation, thereby reducing the risk of several potential infections [143]. Copper and copper alloys can also be used to produce sanitary installation tubes, fittings, door handles, knobs, hand-rails, and vehicle door handles to reduce the microbial burden.

Apart from the above, silver-zinc (Ag-Zn) and silver-copper (Ag-Cu) incorporated soda-lime glass prepared by ion exchange has shown significant antimicrobial properties [147], which could be useful for several daily applications, specifically in the health industry and public places. Copper metal and CuO-NPs embedded in a polypropylene matrix is also an evidence of antimicrobial property. After careful preparation, Delgado et al. [148] found that the composite has a strong antimicrobial activity against *E. coli* and could kill 95% of the colony within four hours, acting through the release of the Cu^2+^ ions.

Although many water purification methods exist, potable water is still beyond the reach of millions of people around the globe. Sudha et al. [149] conducted a study to investigate the microbial efficacy of copper pots in killing the bacteria (*V. cholera* O1, *Shigella flexneri (S. flexneri)* 2a, *E. coli, Salmonella enterica Typhi (S. Typhi),* and *Salmonella enterica serovar Paratyphi (S. Paratyphi*) in the water. When drinking water was contaminated with 500 CFU/mL of those bacteria stored in copper pots for 16 h at room temperature, no bacteria were recovered in the culture medium. They observed a slight alteration in the water pH from 7.83 to 7.93, but no other changes were observed. Copper pots are consequently considered an interim microbial purification solution for drinking water in under-developed and many developing country areas [149]. It is also found that a copper-coiled device put into a glass bottle overnight can also inactivate the bacteria, including *E. coli*, *S. typhi,* and V. *cholera* [150]. This observation still needs to be investigated for the possibility of viable but non-culturable microbial inactivation. It is recalled that the use of copper or copper alloy pots were common in Indian, Chinese, and Egyptian households, but they were displaced by the arrival of cheaper aluminium, steel, and plastic wares. The major advantage of copper’s biocidal activity is that there is no need for any kind of energy, fuel, including electricity. It is also found that similar to dry copper metal efficacy, the biocidal effectiveness of copper in water also follows a temperature pattern, with the fastest biocidal effects occurring at higher temperatures [151]. The presence of chloride salts (NaCl) resulted in faster inactivation (of *E. coli*) compared with pure water, but the presence of complex organic mixtures such as humic acids, proteins, and amino acids reduced the inactivation [151]. It gives an indication that natural organic constituents and salts in the water influence the antibacterial efficacy of copper when used to treat water.

In addition to the above, the antimicrobial efficacy of copper-impregnated textiles and latex were mentioned in many studies [152,153]. For example, research from Israel highlighted the antimicrobial properties of copper-impregnated textiles [152], and the researchers developed copper-impregnated textile products such as cotton and latex and tested them against various bacteria (*E. coli, S. aureus*, MRSA, and VRE), viruses (HIV-1 and West Nile virus), and fungi (*C. albicans*). They demonstrated that >2 log reduction of all tested bacteria occurred within two hours, and the materials inactivated the fungi within an hour. They also observed that latex gloves were able to reduce the infectivity of both viruses (HIV-1 and West Nile virus). Later studies also found that copper oxide-impregnated fabrics could reduce >98.7% of microbes within 20 to 240 min of exposure [153].

## 7. Possible Environmental Impacts Arising from the Use of Copper and Copper Alloys

Even though copper is an essential nutrient for the growth of plants and humans and copper compounds have demonstrated infection control properties and can be used in various medical applications, copper’s environmental impacts should be considered seriously. Despite its comprehensive daily and industrial applications, copper compounds are toxic to fish and several other ecosystems, but the exact effects are largely unknown. Plants are able to uptake copper from the soil surface, which depends on the species, bioavailability, composition, and growth of the media [154]. Gomes et al. [155] investigated the potential toxicity of copper nanoparticles in the gills of mussels *Mytilus galloprovincialis* by exposing the mussels to 10 µg of Cu/L for 15 days. The results revealed that mussels accumulated copper in their gills and have shown different responses to CuO-NPs and Cu^2+^. The CuO-NPs induced oxidative stress in mussels by overwhelming antioxidant defence system, whereas Cu^2+^ increased the enzymatic activities occasionally [155]. It has been observed that excessive copper could induce oxidative stress in plants by inducing reactive oxygen species [156]. The accumulation of CuO-NPs in the roots of hydroponically grown lettuce and alfalfa, and the translocation of Cu-NPs to the stem, leaves, and fruits of cucumbers exposed to Cu-NPs, have been positively identified [157,158], which can reach humans via the food chain mechanism.

Even though nanotechnology is applied in various areas of human lives, several NPs may have the ability to harm the environment and the human body through easy exposure (inhalation, dermal penetration, ocular exposure, and ingestion) due to their very small size [159]. Cu-NPs and CuO-NPs released into the environment, mainly from wastewater treatment facilities and agriculture systems, can reach potable water systems [154,160]. Many studies have warned about NPs’ applications in the human environment as it can deposit both at the upper and lower respiratory tracts and can generate inflammatory responses [161,162,163]. Apart from this, Cu-NPs can mobilise through tissues and can enter into the bloodstream in the body [164]. In humans, excess copper can cause complex toxicological effects such as DNA damage, oxidative stress, including lipid peroxidation, and can instigate Wilsons’ disease [165,166,167]. The high toxic effects in the human body can generate acute symptoms such as nausea, vomiting, and cramping, associated with the gastro-intestinal tract [168], and it is considered that the toxic effects usually occur when the copper concentration is >30 mg/L [169]. Even though several health effects of copper were identified, a hydroponic system study revealed that the co-existence of As(V) with CuO-NPs led to a 45% decrease in As(V) in rice roots [170]. Therefore, more studies are required before the application of Cu-NP-impregnated materials, specifically in the healthcare environment, including their disposal and the disposal of the contaminated water obtained after washing Cu-NP-impregnated cloths and plastics. In order to avoid environmental contamination, it has been recommended to use copper in a chelated form, as this complex is non-reactive with other chemicals in the water [171,172]. An example of this approach with chelates, copper-8-quinolinolate, and its derivatives are widely used in controlling *Aspergillus* fungal infections in hospitals and also in fruit-handling equipment.

Many studies have warned about nanoparticles’ applications in the human environment [161,162], and it is agreed that more studies are required before the wider applications of copper nanoparticle-impregnated materials. This pertains specifically to the healthcare environment and focuses on the post-use strategy with copper nanoparticle-impregnated cloths and plastics, including its washing. Although it is noted that NPs have key uses in the human-affected landscape, benefits need to be assessed relative to risks arising from their production and their disposal [173].

## 8. Antimicrobial Mechanism of Copper

**Figure 3:**
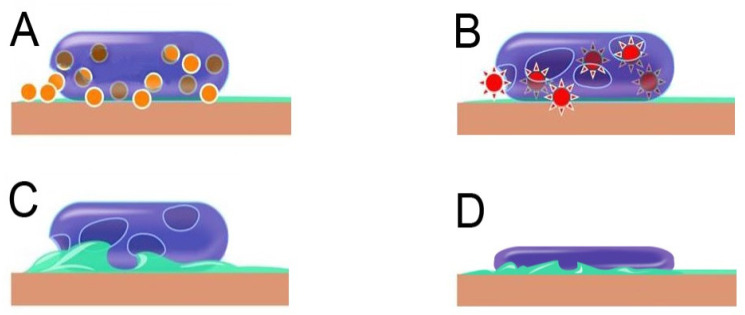
Artist’s view of the critical steps that are considered to be taking place in contact-killing. (**A**) When a bacterium is in touch with the copper or copper alloy surfaces, the free electrons from the copper move into the cell, probably due to the potential difference between the metal and the interior of the bacterium. (**B**) The hitting of the electron generates stress that creates holes in the bacteria’s cell membrane (**C**) When the holes become big enough, the contents in the cytoplasm come out. (**D**) The copper ions induced the generation of reactive oxygen species to cause further cell damage resulting in genomic and DNA degradation (but not in all cases) results in the death of the bacterium. In support of this, it is reported that when *E. coli* cells were applied on copper coupons, a large number of copper ions were taken by *E. coli* within 90 min, and the absorption is found to be more when the bacteria plated on the copper surface by dry method. Adapted with permission from Ref. [3]. Copyright 2007, American Society for Microbiology.

Based on substantial evidence, the antimicrobial property of copper is very complex and difficult to understand [174,175]. This is considered due to the atomic structure of the copper atoms, specifically its ability to easily donate or receive electrons, making copper an excellent electric conductor and potential antimicrobial [176]. When in contact with microbes, the copper’s free electrons can easily interact with the microbial protein and suppress its activity. With respect to the ionic forms of copper, Cu^+^ (cuprous ion) is seen to be more bactericidal than the cupric ion (Cu^2+^) due to its higher penetration ability into the bacterial membranes [2]. The most distinguishing feature of metallic copper or alloyed copper surfaces, including Cu-NPs, is their ability to achieve very high killing efficacy of microbes in close contact within a short time [75,82,95,119,177,178]. This phenomenon, called “contact-killing”, is particularly accelerated under “dry” conditions (a few to several minutes) compared to the wet conditions (several hours) [95,179]. Although several investigations have been carried out on the antimicrobial property of Cu and Cu-NPs, the mechanisms by which microbes are killed when in contact with Cu-containing surfaces and Cu-NPs are not fully understood. It is currently considered that cell destruction caused by the copper ions is the main cause (Figure 3) [3,95]. When *E. coli* was applied to copper surfaces through aqueous suspension, it was found that the *E. coli* cells were filled with a large amount of copper ions within 90 min. When the same application is made with minimum liquid with a drying time of five seconds, the cells’ copper ion accumulation was more dramatic [95]. Another experiment by Santo et al. [180] using *Staphylococcus haemolyticus* also evidenced a large amount of copper ion accumulation in the cell. The elevated number of copper ions during the death of the bacteria in their cells may be enough to suggest that the copper ions cause cell death. However, Weaver et al. [9] independently found that copper kills MRSA by causing DNA damage. At this time, several theories have been proposed to explain the mechanism behind contact-killing, emphasizing the complex nature of this function.

### 8.1. Membrane Depolarisation

Membrane polarisation is considered a mechanism by which copper ions can exert a toxic effect on bacteria [95,177]. It is thought that metabolically active bacteria have an electrical potential difference (~100–200 mV) between the inside and outside of the cell that can vary with species. Generally, the exterior may have high potential compared to the interior. When the copper ions bind to negatively charged domains on the bacteria cell membrane, the potential difference decreases, and depolarisation can occur. When the potential difference reaches zero, membrane weakness or rupture can happen [95,177]. The bindings of copper ions at the peptidoglycans or on the lipopolysaccharide carboxyl groups have also been reported, which may also affect the microbe’s cell envelope stability, but all these postulated mechanisms may depend on the bacteria’s morphology [181,182]. However, Warnes et al. [177] believe that membrane depolarisation is very rapid in some bacteria such as *E. coli* and *Salmonella*, whereas it is slow in the case of *Enterococcus*.

### 8.2. Reactive Oxygen Species Generation

The reactive oxygen species (ROS) hypothesis highlights the impacts of Cu-NPs on bacterial cells [183,184], but it also strongly depends on the copper’s oxidation state. (At the same time, it should be noted that there is a contradictory report has been presented which argues against the ROS hypothesis [185].) This process is explained by the formation of hydroxyl radicals [186]. The addition of ROS potentiality is associated with intermediate sulphur radical chemistry in relation to the reduction of Cu (II) by intracellular thiols [171]. Increased ROS leads to oxidative damage, especially in DNA and membrane lipid peroxidation [167]. The possibility of direct binding of Cu-NPs to bacterial DNA was also proposed [183], but contradictory reports regarding DNA damage exist [75,96,178,185]. It is also reported that copper ions can impair intracellular protein/enzyme activity by the metal catalysed oxidation of amino acid residues, site-specific inactivation of iron-sulphur clusters, and displacement of essential cofactors from metalloenzymes [186], as it is found that copper ions can bind to a membrane-bound enzyme–peptidoglycan LD-transpeptidase, which increased the permeability of the bacterial cell wall [187].

The first event is the dissolution of copper ions from the copper surface and its accumulation on the bacterial membrane or within the cell, which can create membrane damage by depolarization, resulting in the leakage of intracellular components. Further cell damage is proposed by ROS generation, leading to DNA degradation, but there are contradictory findings reported. Warnes and Keevil [188] observed rapid and extensive DNA fragmentation of vancomycin-resistant enterococci when in contact with copper alloy surface, but that cannot happen when the bacteria are in contact with stainless steel. During another study with *enterococci*, the same researchers observed rapid ROS generation and DNA degradation, including respiratory inhibition, leading to cell membrane damage [188]. This is also supported by Li et al. [178]. They mentioned that as a result of the membrane damage, leakage of the cellular materials occurs, followed by the inactivation of the respiratory activity. However, in a later work, they observed immediate membrane depolarisation, but the DNA degradation was much slower in *E. coli* and *S. aureus* when contact with copper alloy surfaces [177]. On the other hand, Santo et al. [95] observed the immediate killing of *E. coli* when in contact with metallic copper surfaces by immediate membrane damage, but they did not observe any DNA damage by either mutation or fragmentation even after cell death [95]. Simultaneously, genetic toxicity is involved, and copper affects gene replication efficiency [178]. This mechanism also resembles that hypothesized by Grass et al. [3] and Selvamani et al. [20]. Thus, it can be inferred that copper’s surface-mediated toxicity may involve membrane depolarization, ROS generation, and DNA degradation along with possible inactivation of respiratory activity, but the exact contact-killing mechanism is expected to be dependent on specific factors such as bacterial morphology or environment conditions such as the presence of moisture or buffering agents. Even though many studies proposed several ideas behind the antimicrobial mechanism of copper and most of the mechanisms are known, there is no consensus found among the researchers on the sequential events, so more studies are required to elucidate the exact mechanism.

Even though only few studies have been carried out to investigate the antimicrobial mechanism of copper on fungi, it is unanimously accepted that the mechanism happening in fungi is similar to that in bacteria. For example, the study of Quaranta et al. [117] on *C. albicans* and *S. cerevisiae* cells on copper coupons (C11000, 99.9% Cu and C75200, 62% Cu) revealed that the first damage was localised on membranes similar to that on bacteria.

Similar to fungi, not many studies were conducted to investigate the antiviral mechanism, primarily because of the difficulty in the study due to the very small size compared to bacteria. The study of Warnes and Keevil [90] proposed that copper targets the viral genome, specifically the gene encoding VPg (viral-genome-protein-linked, a viral protein essential for viral inactivity) by gene copy number reduction. Later, Warnes et al. [93] confirmed this result.

Apart from copper, silver also shows antimicrobial efficacy. It is the most extensively studied metal since it has been used for centuries, especially in various medical applications, including coatings for medical catheters and implants and wound dressings [189,190]. Even though metallic silver was showing good antimicrobial activity for centuries, particularly in the Greek civilization, its use declined in 1937 as it was recognised that the contact-killing power of silver was not great as expected [191]. However, silver nanoparticles (Ag-NPs) still provide excellent antimicrobial service, although later research identified that copper has superiority over silver surfaces for contact-killing [184]. Through a recent study, Luo et al. [192] understood that silver does not possess any antimicrobial activity during the first six hours of exposure to microbes, and only a marginal contact-killing potential (<1 log) occurs within the next three hours. A “touch transfer assay” modelling fingerprint transmission was developed by Knobloch et al. [193] that can test the antimicrobial efficacy of any surfaces under realistic indoor conditions. When tested with silver surfaces, no significant bacteria reduction was seen within the first 24 h, while copper surfaces displayed a minimum of 2 log reduction of the bacterial load [193]. The study shows that silver required fluid presence or high moisture content to generate silver ions necessary for the contact-killing, whereas copper does not require moisture to generate ions [194]. Another comparative study of silver and copper surfaces in typical laboratory conditions (22 °C and 50% relative humidity Cu11000 surfaces, which are 99.9% Cu), exhibited a 7 log reduction of bacteria within 75 min of exposure, whereas silver could not destroy considerable numbers of bacteria even up to 360 min [75]. As a result of these studies and considering the lower cost of copper than silver, copper is the desired choice for contact-killing of microbes that can be used in indoor and outdoor environments. In lieu of the copper oxide formation, many may doubt the long-term effect of copper surfaces, particularly in the outdoor environment; however, studies show that the copper oxide formation does not affect copper’s contact-killing ability [195].

## 9. The Economics of Copper Installation and Advantages?

We have discussed that there are several methods of copper application available to reduce the bioburden and transmission in public areas, such as the use of copper products, installation of copper surfaces over frequently touched areas, installation of microfilms, and the use of copper nanoparticle-impregnated clothes and personal protective equipment(s) (PPEs) in the health and food industries. Apart from these applications, the formation of three copper surfaces produced by the deposition by different methods of copper sprays was studied by Champagne et al. [196]. The authors used three modes of copper spray, namely plasma, wire-arc, and cold sprays, to investigate the efficacy of each against MRSA. They found that the cold spray method had much more significant effects when compared to the other two methods.

We generally think that the installation of copper products and surfaces is very expensive, but in reality, such copper installations and material provisions are relatively inexpensive compared to the potential treatment cost associated with HAIs. The cost estimates involved with such work were closely studied by Michels et al. [27], who reported that the installation of the copper surface in the healthcare environment is economically profitable compared to subsequent HAI patient treatment costs. Based on their study, the structure of copper surfaces reduced the total medical situation within the experimental area by 14 infections over 338 days. According to the Centres for Disease Control, the average cost of treating an HAI patient varies from USD 28.4K to USD 33.8K [197]. The cost of installing the copper surface, according to the experiment conducted by Michels et al. [27], is USD 52K. Thus, if the copper surface can prevent 14 infections, it can save a minimum of USD 397.6K over 338 days (USD 1176 per day). Additionally, it could be commented that, since alloys function in a similar way to pure copper, the production cost of copper materials and copper surfaces could be significantly reduced.

Recent studies have found that the provision of copper or copper alloy surfaces in the healthcare environment can reduce HAIs up to 58%, and this evidence indicates that serious consideration is warranted for the introduction of copper surfaces when addressing infection reduction in health and food industries in public places and on public transport. It is also seen that Cu-NPs and laser textured copper surfaces are very effective control measures, and the introduction of these materials into cheaper touch surfaces should be urgently considered. The advantages of copper are, but are not limited to:

(i) It is easy and safe to install and maintain and is visually attractive;
(ii) Whist the initial installation expenditure appears to be high, it gives excellent return when compared to otherwise required treatment expenditure;
(iii) Once installed, copper products and surfaces continue to provide non-diminishing infection control;
(iv) There is no requirement for energy input, apart from regular cleaning, needed for maintaining the antimicrobial properties;
(v) Copper products and surfaces do not introduce any harmful side effects to the health when installed in public places; and
(vi) It is a convenient and effective way to control superbugs such as MRSA and VRE.

## 10. Conclusions

The transmission of microbial pathogens through touching contaminated surfaces is always a significant and concerning problem for public health authorities. Studies have shown that the use of disinfecting methods to reduce the accumulation and transmission of the bioburden, although important, is not completely successful. This paper has argued that the modification of fomites by providing pure copper, copper alloys, and copper-impregnated products on frequent touch surfaces in the health and food industries and public places may make a considerable difference to the community and individual health outcomes. Research and development in this area of touch surfaces have lagged for some years because of the previous wide availability of effective antibiotics, but serious problems have emerged as a result of new antibiotic-resistant pathogens, plus the emergence of hitherto unknown strains such as SARS-CoV-2.

The adage “prevention is better than cure” is widely agreed to be the best advice to follow from a public health perspective. Once an infection occurs and disease manifests in the body, notwithstanding the fact that there are remarkable possibilities for treatment in the healthcare environment, avoidance of severe consequences cannot always be guaranteed. This is particularly so in a situation like COVID-19, especially when patients are aged and have many types of comorbidities. In addition, even whilst treatment may be successful, the trauma may create lasting physical and mental issues, time is lost away from work and studies, and significant losses of savings can occur. Therefore, preventing the catching of an infection and avoiding the subsequent disease is arguably the best option to maintain health and well-being. From this perspective, we suggest that copper products and the installation of copper surfaces in hospitals and other healthcare facilities, food industries, public places, and public transport have a significant role in fostering community health by infection reduction and prevention.

Communities are currently practicing a range of infection control procedures during this time of the COVID-19 pandemic. Hand hygiene, regular surface cleaning with disinfectants, applications of UV radiation, and infusion of hydrogen peroxide mists have all been investigated. However, many of these methods are not practical for large public gathering places and on public transport, and it is here that the effect of copper will be advantageous as it has continuing self-sanitising properties. Since copper products and surfaces act as a reducer or barrier to touch-transferred infections, the use of copper products and the installation of copper or copper alloy surfaces in healthcare facilities, public places, and public transport may reduce or avoid many of the current and upcoming infectious diseases.

## Figures and Tables

**Table 1:** Showing the implications of healthcare-associated or hospital-acquired infections (HAIs) in the USA, the UK, Europe, and Australia, including the annual number of registered infections, the number of mortalities per annum, and the annual expenditure calculated.

Sl. No.	Country	No of Cases Per Annum	No of Mortalities Per Annum	Expenditure Per Annum (USD)	Reference(s)
1	USA	687,000	72,000	35 to 45 billion	[28]
2	Europe	3.8 million	90,000	8.3 billion	[31]
3	England	834,000	28,500	3.71 billion	[33]
4	Australia	165,000	Unknown	4.7 billion	[34,35,36]

## Data Availability

Not applicable.

## References

[1] Borkow G., Gabbay J. (2019). Copper, an ancient remedy returning to fight microbial, fungal and viral infections. Curr. Chem. Biol..

[2] Chaturvedi K.S., Henderson J.P. (2014). Pathogenic adaptations to host-derived antibacterial copper. Front. Cellu. Infect. Micobiol..

[3] Grass G., Rensing C., Solioz M. (2011). Metallic copper as an antimicrobial surface. Appl. Environ. Microbiol..

[4] Dollwet H., Sorenson J.R.J. (1985). Historic uses of copper compounds in medicine. Trace Elem. Med..

[5] Carson K.C., Bartlett J.G., Tan T.J., Riley T.V. (2007). In vitro susceptibility of methicillin-resistant Staphylococcus aureus and methicillin-susceptible Staphylococcus aureus to a new antimicrobial, copper silicate. Antimicrob. Agents Chemother..

[6] Ruparelia J.P., Chatterjee A.K., Duttagupta S.P., Mukherji S. (2008). Strain specificity in antimicrobial activity of silver and copper nanoparticles. Acta Biomater..

[7] Ren G., Hu D., Cheng E.W., Vargas-Reus M.A., Reip P., Allaker R.P. (2009). Characterisation of copper oxide nanoparticles for antimicrobial applications. Int. J. Antimicrob. Agent.

[8] Stout J.E., Murder R.R., Mietzner S., Wagner M.M., Perri M.B., DeRoss K., Goodrich D., Arnold W., Theresa Williamson T., Ruark O. (2007). Role of environmental surveillance in determining the risk of hospital-acquired legionellosis: A national surveillance study with clinical correlations. Infect. Contr. Hosp. Epidemiol..

[9] Weaver L., Michels H.T., Keevil C.W. (2010). Potential for preventing spread of fungi in air-conditioning systems constructed using copper instead of aluminium. Lett. Appl. Microbiol..

[10] Fujimori Y., Sato T., Hayata T., Nagao T., Nakayama M., Nakayama T., Sugamata R., Suzuki K. (2012). Novel antiviral characteristics of nanosized copper (I) iodide particles showing inactivation activity against 2009 pandemic H1N1 influenza virus. Appl. Environ. Microbiol..

[11] Olivares M., Uauy R. (1996). Copper as an essential nutrient. Am. J. Clin. Nutr..

[12] Barceloux D.G., Barceloux D. (1999). Copper. J. Toxicol Clini. Toxicol.

[13] Brewer G.J. (2003). Copper in medicine. Curr. Opin. Chem. Biol..

[14] O’Brien P.A., Kulier R., Helmerhorst F.M., Usher-Patel M., d’Arcangues C. (2008). Copper-containing, framed intrauterine devices for contraception: A systematic review of randomized controlled trials. Contraception.

[15] Michels H.T., Anderson D.G., Collery P., Marymard I., Theophanides T., Khassanova L., Collery T. (2008). Antimicrobial regulatory efficacy testing of solid copper alloy surfaces in the USA. Met Ions Biology and Medicine.

[16] Schmidt M.G., Attaway H.H., Sharpe P.A., John J., Sepkowitz K.A., Morgan A., Fairey S.E., Singh S., Steed L.L., Cantey J.R. (2012). Sustained reduction of microbial burden on common hospital surfaces through the introduction of copper. J. Clin. Microbiol..

[17] Schmidt M.G., Attaway H.H., Fairey S.E., Howard J., Mohr D., Craig S. (2019). Self-disinfecting copper beds sustain terminal cleaning and disinfection effects throughout patient care. Appl. Environ. Microbiol..

[18] Lazary A., Weinberg I., Vatine J.-J., Jefidoff A., Bardenstein R., Borkow G., Ohana N. (2014). Reduction of healthcare-associated infections in a long-term care brain injury ward by replacing regular linens with biocidal copper oxide impregnated linens. Int. J. Infect. Dis..

[19] Niiyama N., Sasahara T., Mase H., Abe M., Saito H., Katsuoka K. (2013). Use of copper alloy for preventing transmission of methicillin-resistant Staphylococcus aureus contamination in the dermatology ward. Acta Derm. Venereol..

[20] Selvamani V., Zareeri A., Elkashiff A., MAruthamuthu M.K., Chittiboyina S., Delisi D., Li Z., Cai L., Pol V.G., Seleem M.N. (2020). Hierarchical micro/mesoporous copper structure with enhanced antimicrobial property via laser surface texturing. Adv. Mater. Interfaces.

[21] Hinsa-Leasure S.M., Nartey Q., Vaverka J., Schmidt M.G. (2016). Copper alloy surfaces sustain terminal cleaning levels in a rural hospital. Am. J. Infect. Control.

[22] Dietz L., Horve P.F., Coil D.A., Fretz M., Eisen J.A., Van Den Wymelenberg K. (2020). 2019 novel coronavirus (COVID-19) pandemic: Built environment considerations to reduce transmission. Msystems.

[23] Sattar S.A. (2001). Survival of microorganisms on animate and inanimate surfaces and their disinfection. Proceedings of the Disinfection, sterilization and antisepsis: Principles and practices in healthcare facilities.

[24] Reynolds K.A., Watt P.M., Boone S.A., Gerba C.P. (2005). Occurrence of bacteria and biochemical markers on public surfaces. Int. J. Environ. Health Res..

[25] Casey A.L., Adams D., Karpanen T.J., Lambert P.A., Cookson B.D., Nightyngale P., Miruszenko L., Shillam R., Christian P., Elliot T.S.J. (2010). Role of copper in reducing hospital environment contamination. J. Hosp. Infect..

[26] Haque M., Sartelli M., McKimm J., Bakar M.A. (2018). Health care-associated infections–an overview. Infect. Drug Resist..

[27] Michels H.T., Keevil C.W., Salgado C.D., Schmidt M.G. (2015). From laboratory research to a clinical trial: Copper alloy surfaces kill bacteria and reduce hospital-acquired infections. HERD: Health Environ. Resear. Design J..

[28] CDC (2018). Data Portal, Healthcare Associated Infections (HAI), Centre for Disease Control (CDC), USA. https://www.cdc.gov/hai/data/portal/index.html.

[29] WHO (2011). Patient Safety. Report on the Burden of Endemic Health Care-Associated Infection Worldwide. https://apps.who.int/iris/bitstream/handle/10665/80135/9789241501507_eng.pdf;jsessionid=9680B80D81E0C4E7C2348B5769668CD0?sequence=1.

[30] ECDPC Healthcare-Associated Infections—A Threat to Patient Safety in Europe. https://www.ecdc.europa.eu/en/publications-data/infographic-healthcare-associated-infections-threat-patient-safety-europe2018.

[31] OECD (2018). Healthcare-associated infections. Health at a Glance: Europe 2018: State of Health in the EU Cycle, Organisation of Economic Cooperation and Development.

[32] Salgado C.D., Sepkowitz K.A., John J.F., Cantey J.R., Attaway H.H., Freeman K.D., Sharpe P.A., Michels H.T., Schmidt M.G. (2013). Copper surfaces reduce the rate of healthcare-acquired infections in the intensive care unit. Infect. Control Hosp. Epidemiol..

[33] Guest J.F., Keating T., Could D., Wigglesworth N. (2020). Modelling the annual NHS costs and outcomes attributable to healthcare-associated infections in England. BMJ.

[34] HAI Healthcare-Associated Infections, Hospital-Acquired Complication-3, Australian Commission on Safety and Quality in Health Care. https://www.safetyandquality.gov.au/sites/default/files/migrated/Healthcare-associated-infection-detailed-fact-sheet.pdf.

[35] Mitchell B.G., Shaban R.Z., MacBeth D., Wood C.J., Russo P.L. (2017). The burden of healthcare-associated infection in Australian hospitals: A systematic review of the literature. Infect. Dis. Health.

[36] Allen J., Rey-Conde T., North J.B., Kruger P., Babidge W.J., Wysocki A.P., Ware R.S., Veerman J.L., Maddern G.J. (2018). Processes of care in surgical patients who died with hospital-acquired infections in Australian hospitals. J. Hosp. Infect..

[37] WHO Food Safety. https://www.who.int/news-room/fact-sheets/detail/food-safety.

[38] CDC Estimates of foodborne illness in the United States. https://www.cdc.gov/foodborneburden/index.html.

[39] Hoffmann S., Maculloch B., Batz M. Economic Burden of Major Foodborne Illness Acquired in the United States. https://www.ers.usda.gov/webdocs/publications/43984/52807_eib140.pdf.

[40] World Bank The Safe Food Imperative: Accelerating Progress in Low and Middle-Income countries. https://www.worldbank.org/en/topic/agriculture/publication/the-safe-food-imperative-accelerating-progress-in-low-and-middle-income-countries.

[41] Boone S.A., Gerba C.P. (2007). Significance of fomites in the spread of respiratory and enteric viral disease. Appl. Environ. Microbiol..

[42] Heikkinen T., Järvinen A. (2003). The common cold. Lancet.

[43] Yezli S., Khan A. (2020). COVID-19 social distancing in the Kingdom of Saudi Arabia: Bold measures in the face of political, economic, social and religious challenges. Trav. Med. Infect. Dis..

[44] Wildman W.J., Bulbulia J., Sosis R., Schjoedt U. (2020). Religion and COVID-19 pandemic. Relig. Brain Behav..

[45] Rawlinson S., Ciric L., Cloutman-Green E. (2020). COVID-19 pandemic—Let’s not forget surfaces. J. Hosp. Infect..

[46] Riddell S., Goldie S., Hill A., Eagles D., Drew T.W. (2020). The effect of temperature on persistence of SARS-CoV-2 on common surfaces. Virol. J..

[47] Andersson D.I., Nicoloff H., Hjort K. (2019). Mechanisms and clinical relevance of bacterial heteroresistance. Nat. Rev. Microbiol..

[48] Majno G. (1991). The Healing Hand: Man and Wound in the Ancient World.

[49] Rogers J.U.L.I.E., Keevil C.W. (1992). Immunogold and fluorescein immunolabelling of Legionella pneumophila within an aquatic biofilm visualized by using episcopic differential interference contrast microscopy. Appl. Environ. Microbiol..

[50] Arendsen L.P., Thakar R., Bassett P., Sultan A.H. (2019). The use of copper as an antimicrobial agent in health care, including obstetrics and gynecology. Clin. Microbiol. Rev..

[51] Van Doremalen N., Bushmaker T., Morris D.H., Holbrook M.G., Gamble A., Williamson B.N., Tamin A., Harcourt J.L., Thornburg N.J., Gerber S.I. (2020). Aerosol and surface stability of SARS-CoV-2 as compared with SARS-CoV-1. NEJM.

[52] Michels H.T., Michels C.A. (2016). Copper alloys-The new ‘old’ weapon in the fight against infectious disease. Microbiology.

[53] Ibrahim Z., Petrusan A.J., Hooke P., Hinsa-Leasure S.M. (2018). Reduction of bacterial burden by copper alloys on high-touch athletic center surfaces. Am. J. Infect. Control.

[54] Wagenvoort J.H.T., Penders R.J.R. (1997). Long-term in-vitro survival of an epidemic MRSA phage-group III-29 strain. J. Hosp. Infect..

[55] Hammett E. (2020). How long does Coronavirus survive on different surfaces?. BDJ Team.

[56] Kuehnert M.J., Hill H.A., Kupronis B.A., Tokars J.I., Solomon S.L., Jernigan D.B. (2005). Methicillin-resistant–Staphylococcus aureus hospitalizations, United States. Emerg. Infect. Dis..

[57] Schmidt M.G., Attaway H.H., Fairey S.E., Steed L.L., Michels H.T., Salgado C.D. (2013). Copper continuously limits the concentration of bacteria resident on bed rails within the ICU. Infect. Control Hosp. Epidemiol..

[58] Różańska A., Chmielarczyk A., Romaniszyn D., Majka G., Bulanda M. (2018). Antimicrobial effect of copper alloys on Acinetobacter species isolated from infections and hospital environment. Antimicrob. Resist. Infect. Control.

[59] Mehtar S., Wiid I., Todorov S.D. (2008). The antimicrobial activity of copper and copper alloys against nosocomial pathogens and Mycobacterium tuberculosis isolated from healthcare facilities in the Western Cape: An in-vitro study. J. Hosp. Infect..

[60] Weaver L., Noyce J.O., Michels H.T., Keevil C.W. (2010). Potential action of copper surfaces on meticillin—Resistant Staphylococcus aureus. J. Appl. Microbiol..

[61] Karpanen T.J., Casey A.L., Lambert P.A., Cookson B.D., Nightingale P., Miruszenko L., Elliot T.S.J. (2012). The antimicrobial efficacy of copper alloy furnishing in the clinical environment: A crossover study. Infect. Control Hosp. Epidemiol..

[62] ECI Copper—A New Weapon to Fight the Influenza a Virus- New Research Finds Copper Effective at Inactivating H1N1 Virus. https://pr.euractiv.com/pr/copper-new-weapon-fight-influenza-virus-new-research-finds-copper-effective-inactivating-h1n1.

[63] Barker J., Vipond I.B., Bloomfield S.F. (2014). Effects of cleaning and disinfection in reducing the spread of Norovirus contamination via environmental surfaces. J. Hosp. Infect..

[64] Kuhn P.J. (1983). Doorknobs: A source of nosocomial infection. Diagn. Med..

[65] Colin M., Klingelschmitt F., Charpentier E., Josse J., Kanagaratnam L., Champs C.D., Gangloff S.C. (2018). Copper alloy touch surfaces in healthcare facilities: An effective solution to prevent bacterial spreading. Materials.

[66] Mikolay A., Huggett S., Tikana L., Grass G., Braun J., Nies D.H. (2010). Survival of bacteria on metallic copper surfaces in a hospital trial. Appl. Microbiol. Biotechnol..

[67] Efstathiou P. (2011). The Role of Antimicrobial Copper Surfaces in Reducing Healthcare-Associated Infections. Eur. Infect. Dis..

[68] O’gorman J., Humphreys H. (2012). Application of copper to prevent and control infection. Where are we now?. J. Hosp. Infect..

[69] von Dessauer B., Navarrete M.S., Benadof D., Benavente C., Schmidt M.G. (2016). Potential effectiveness of copper surfaces in reducing health care–associated infection rates in a pediatric intensive and intermediate care unit: A nonrandomized controlled trial. Am. J. Infect. Control.

[70] Schmidt M.G., Dessauer B.v., Benavente C., Benadof D., Cifuentes P., Elgueta A., Duran C., Navaratte M.S. (2016). Copper surfaces are associated with significantly lower concentrations of bacteria on selected surfaces within a paediatric intensive care unit. Am. J. Infec. Control.

[71] Zhu L., Elguindi J., Rensing C., Ravishankar S. (2012). Antimicrobial activity of different copper alloy surfaces against copper resistant and sensitive Salmonella enterica. Food Microbiol..

[72] Souli M., Antoniadou A., Katsarolis I., Mavrou I., Paramythiotou E., Papadomichelakis E., Drogari-Apiranthitou M., Panagea T., Giamarellou H., Petrikkos G. (2017). Reduction of environmental contamination with multidrug-resistant bacteria by copper-alloy coating of surfaces in a highly endemic setting. Infect. Control Hosp. Epidemiol..

[73] Weaver L., Michels H.T., Keevil C.W. (2008). Survival of Clostridium difficile on copper and steel: Futuristic options for hospital hygiene. J. Hosp. Infect..

[74] Elguindi J., Wagner J., Rensing C. (2009). Genes involved in copper resistance influence survival of Pseudomonas aeruginosa on copper surfaces. J. Appl. Microbiol..

[75] Michels H.T., Noyce J.O., Keevil C.W. (2009). Effects of temperature and humidity on the efficacy of methicillin—Resistant Staphylococcus aureus challenged antimicrobial materials containing silver and copper. Appl. Microbiol..

[76] Noyce J.O., Michels H., Keevil C.W. (2006). Potential use of copper surfaces to reduce survival of epidemic methicillin-resistant Staphylococcus aureus in the healthcare environment. J. Hosp. Infect..

[77] Noyce J.O., Michels H., Keevil C.W. (2006). Use of copper cast alloys to control Escherichia coli O157 cross-contamination during food processing. Appl. Environ. Microbiol..

[78] Wilks S.A., Michels H., Keevil C.W. (2005). The survival of Escherichia coli O157 on a range of metal surfaces. Int. J. Food Microbiol..

[79] Wilks S.A., Michels H.T., Keevil C.W. (2006). Survival of Listeria monocytogenes Scott A on metal surfaces: Implications for cross-contamination. Int. J. Food Microbiol..

[80] Bleichert P., Santo C.E., Hanczaruk M., Meyer H., Grass G. (2014). Inactivation of bacterial and viral biothreat agents on metallic copper surfaces. Biometals.

[81] Inkinen J., Mäkinen R., Keinänen-Toivola M.M., Nordström K., Ahonen M. (2017). Copper as an antibacterial material in different facilities. Lett. Appl. Microbiol..

[82] Wheeldon L.J., Worthington T., Lambert P.A., Hilton A.C., Lowden C.J., Elliott T.S.J. (2008). Antimicrobial efficacy of copper surfaces against spores and vegetative cells of Clostridium difficile: The germination theory. J. Antimicrob. Chemother..

[83] Goudarzi M., Saviz S., Ghoranneviss M., Salar Elahi A. (2017). Medical equipment antiseptic processes using the atmospheric plasma sprayed copper coatings. J. X-ray Sci. Technol..

[84] Silk B.J., Mahon B.E., Griffin P.M., Gould L.H., Tauxe R.V., Crim S.M., Jackson K.A. (2013). Vital signs: Listeria illnesses, deaths, and outbreaks—United States, 2009–2011. MMWR. Morb. Mortal. Wkly. Rep..

[85] Zhu Q., Gooneratne R., Hussain M.A. (2017). Listeria monocytogenes in fresh produce: Outbreaks, prevalence and contamination levels. Foods.

[86] Wei P., Bao R., Fan Y. (2020). Brainstem Encephalitis Caused by Listeria monocytogenes. Pathogens.

[87] Aisha A. (2005). Antimicrobial Effects of Copper and Brass Ions on the Growth of Listeria Monocytogenes at Different Temperatures, pH and Nutrients. http://www.openthesis.org/documents/Antimicrobial-Effects-Copper-Brass-Ions-526730.html.

[88] Brennan S., McDonald S., McKenzie J., Cheng A., Green S., Allen K. (2017). Systematic review of antimicrobial surfaces to reduce infection rates in hospitalized populations. Cochrane Aust..

[89] Bauer D.J. (1958). The chemotherapeutic activity of compounds of copper, rhodium and certain other metals in mice infected with neurovaccinia and ectromelia viruses. Br. J. Exp. Pathol..

[90] Warnes S.L., Keevil C.W. (2013). Inactivation of norovirus on dry copper alloy surfaces. PLoS ONE.

[91] Science Daily Copper Could Prevent the Spread of Flu Infections (14 February 2006). https://www.sciencedaily.com/releases/2006/02/060214080834.htm.

[92] Noyce J.O., Michels H., Keevil C.W. (2007). Inactivation of influenza a virus on copper versus stainless steel surfaces. Appl. Environ. Microbiol..

[93] Warnes S.L., Little Z.R., Keevil C.W. (2015). Human coronavirus 229E remains infectious on common touch surface materials. MBio.

[94] Cordis Copper Could Stop Spread of Flu. https://cordis.europa.eu/article/id/25151-copper-could-stop-spread-of-flu.

[95] Santo C.S., Lam E.W., Elowsky C.G., Quaranta D., Domaille D.W., Chang C.J., Grass G. (2011). Bacterial killing by dry metallic copper surfaces. Appl. Environ. Microbiol..

[96] Warnes S.L., Green S.M., Michels H.T., Keevil C.W. (2010). Biocidal efficacy of copper alloys against pathogenic enterococci involves the degradation of genomic and plasmid DNAs. Appl. Environ. Microbiol..

[97] Michels H., Moran W., Michel J. (2008). Antimicrobial properties of copper alloy surfaces, with a focus on hospital-acquired infections. Int. J. Met..

[98] Borkow G., Lara H.H., Covington C.Y., Nyamathi A., Gabbay J. (2008). Deactivation of human immunodeficiency virus type 1 in medium by copper oxide-containing filters. Antimicrob. Agents Chemother..

[99] Minoshima M., Lu Y., Kimura T., Nakano R., Ishiguro H., Kubota Y., Hashimoto K., Sunada K. (2016). Comparison of the antiviral effect of solid-state copper and silver compounds. J. Hazard Mater..

[100] Hang X., Peng H., Song H., Qi Z., Miao X., Xu W. (2015). Antiviral activity of cuprous oxide nanoparticles against hepatitis C virus in vitro. J. Virol. Methods.

[101] Chauhan G., Rath G., Goyal A.K. (2013). In-vitroanti-viral screening and cytotoxicity evaluation of copper-curcumin complex. Artif. Cells Nanomed. Biotech..

[102] Shionoiri N., Sato T., Fujimori Y., Nakayama T., Nemoto M., Matsunaga T., Tanaka T. (2012). Investigation of the antiviral properties of copper iodide nanoparticles against feline calicivirus. J. Biosci. Bioengin..

[103] ICA The International Copper Association. http://www.copperinfo.com.

[104] Sagripanti J.L., Routson L.B., Bonifacino A.C., Lytle C.D. (1997). Mechanism of copper-mediated inactivation of herpes simplex virus. Antimicrob. Agents Chemother..

[105] Borkow G., Gabbay J. (2005). Copper as a biocidal tool. Curr. Med. Chem..

[106] Cha J.S., Cooksey D.A. (1991). Copper resistance in Pseudomonas syringae mediated by periplasmic and outer membrane proteins. Proc. Natl. Acad. Sci. USA.

[107] Cooksey D.A. (1993). copper uptake and resistance in bacteria. Mol. Microbiol..

[108] Kumbhar A.S., Padhye S.B., Saraf A.P., Mahajan H.B., Chopade B.A., West D.X. (1991). Novel broad-spectrum metal-based antifungal agents. Biol. Met..

[109] Mumcuoglu K.Y., Gabbay J., Borkow G. (2008). Copper oxide-impregnated fabrics for the control of house dust mites. Int. J. Pest Manag..

[110] Bellí N., Marín S., Sanchis V., Ramos A.J. (2006). Impact of fungicides on Aspergillus carbonarius growth and ochratoxin A production on synthetic grape-like medium and on grapes. Food Addit. Contam..

[111] La Torre A., Iovino V., Caradonia F. (2018). Copper in plant protection: Current situation and prospects. Phytopathol. Mediterr..

[112] Ballo M.K., Rtimi S., Kiwi J., Pulgarin C., Entenza J.M., Bizzini A. (2017). Fungicidal activity of copper-sputtered flexible surfaces under dark and actinic light against azole-resistant Candida albicans and Candida glabrata. J. Photochem. Photobiol. B Biol..

[113] Creaven B.S., Duff B., Egan D.A., Kavanagh K., Rosair G., Thangella V.R., Waslh M. (2010). Anticancer and antifungal activity of copper (II) complexes of quinolin-2 (1H)-one-derived Schiff bases. Inorg. Chim. Acta.

[114] Ghasemian E., Naghoni A., Tabaraie B., Tabaraie T. (2012). In vitro susceptibility of filamentous fungi to copper nanoparticles assessed by rapid XTT colorimetry and agar dilution method. J. Mycol. Médicale.

[115] Usman M.S., El Zowalaty M.E., Shameli K., Zainuddin N., Salama M., Ibrahim N.A. (2013). Synthesis, characterization, and antimicrobial properties of copper nanoparticles. Int. J. Nanomed..

[116] Kruk T., Szczepanowicz K., Stefańska J., Socha R.P., Warszyński P. (2015). Synthesis and antimicrobial activity of monodisperse copper nanoparticles. Colloids Surf. B Biointerfaces.

[117] Quaranta D., Krans T., Santo C.S., Elowsky C.G., Domaille D.W., Chang C.J., Grass G. (2011). Mechanisms of contact-mediated killing of yeast cells on dry metallic copper surfaces. Appl. Environ. Microbiol..

[118] Molteni C., Abicht H.K., Solioz M. (2010). The killing of bacteria by copper surfaces involves dissolved copper. Appl. Environ. Microbiol..

[119] Santo C.E., Morais P.V., Grass G. (2010). Isolation and characterization of bacteria resistant to metallic copper surfaces. Appl. Environ. Microbiol..

[120] Faúndez G., Troncoso M., Navarrete P., Figueroa G. (2004). Antimicrobial activity of copper surfaces against suspensions of Salmonella enterica and Campylobacter jejuni. BMC Microbiol..

[121] Remyadevi J., Jeyasubramanian K., MArikani A., Rajkumar G., Rahuman A.A. (2012). Synthesis and antimicrobial activity of copper nanoparticles. Mater. Lett..

[122] Balela M.D.L., Amores K.L.S. (2019). Electroless deposition of copper nanoparticles for antimicrobial coating. Mater. Chem. Phys..

[123] Raffi M., Hussain F., Bhatti T.M., Akhter J.I., Hameed A., Hasan M.M. (2010). Investigations into the antibacterial behaviour of copper nanoparticles against Escherichia coli. Ann. Microbiol..

[124] Anita S., Ramachandran T., Rajendran R., Koushik C.V., Mahalakshmi M.A. (2011). Study of the antimicrobial property of encapsulated copper oxide nanoparticles on cotton fabric. Text. Res. J..

[125] Harikumar P.S., Aravind A. (2016). Antebacterial activity of copper nanoparticles and nanocomposites against Escherichia coli bacteria. Int. J. Sci..

[126] Palza H., Nuñez M., Bastías R., Delgado K. (2018). In situ antimicrobial behavior of materials with copper-based additives in a hospital environment. Int. J. Antimicrob. Agents..

[127] Applerot G., Lellouche J., Lipovsky A., Nitzan Y., Lubart R., Gedanken A., Banin E. (2012). Understanding the antibacterial mechanism of CuO nanoparticles: Revealing the route of induced oxidative stress. Small.

[128] Giannousi K., Lafazanis K., Arvanitidis J., Pantazaki A., Dendrinou-Samara C. (2014). Hydrothermal synthesis of copper-based nanoparticles: Antimicrobial screening and interaction with DNA. J. Inorg. Biochem..

[129] Dizaj S.M., Mennati A., Jafari S., Khezri K., Adibkia K. (2015). Antimicrobial activity of carbon-based nanoparticles. Adv. Pharma. Bull..

[130] Padil V.V.T., Černík M. (2013). Green synthesis of copper oxide nanoparticles using gum karaya as a biotemplate and their antibacterial application. Int. J. Nanomed..

[131] Heinlaan M., Ivask A., Blinova I., Dubourguier H.C., Kahru A. (2008). Toxicity of nanosized and bulk ZnO, CuO and TiO_2_ to bacteria Vibrio fischeri and crustaceans Daphnia magna and Thamnocephalus platyurus. Chemosphere.

[132] Griffitt R.J., Weil R., Hyndman K.A., Denslow N.D., Powers K., Taylor D., Barber D.S. (2009). Exposure to copper nanoparti-cles causes gill injury and acute lethality in zebrafish (Danio rerio). Environ. Sci. Technol..

[133] Chen Z., Meng H.A., Xing G.M., Chen C.Y., Zhao Y.L., Jia G.A., Wang T.C., Yuan H., Ye C., Zhao F. (2006). Acute toxicological effects of copper nanoparticles in vivo. Toxicol. Lett..

[134] Edwards M., Sprague N. (2001). Organic matter and copper corrosion by-product 455 release: A mechanistic study. Corros. Sci..

[135] Sabrià M., Garcia-Nunez M., Pedro-Botet M.L., Sopena N., Gimeno J.M., Reynaga E., Morera J., Rey-Joly C. (2001). Presence and chromosomal subtyping of Legionella species in potable water systems in 20 hospitals of Catalonia, Spain. Infect. Control Hosp. Epidemiol..

[136] Cunha B.A., Burillo A., Bouza E. (2016). Legionnaires’ disease. Lancet.

[137] Sabria M., Victor L.Y. (2002). Hospital-acquired legionellosis: Solutions for a preventable infection. Lancet Infect. Dis..

[138] Stout J.E., Victor L.Y. (2003). Experiences of the first 16 hospitals using copper-silver ionization for Legionella control: Implications for the evaluation of other disinfection modalities. Infect. Control Hosp. Epidemiol..

[139] Cachafeiro S.P., Naveira I.M., García I.G. (2007). Is copper–silver ionisation safe and effective in controlling legionella?. J. Hosp. Infect..

[140] Casari E., Ferrario A., Montanelli A. (2007). Prolonged effect of two combined methods for Legionella disinfection in a hospital water system. Ann. Ig. Med. Prev. ComunitÃ.

[141] Chen Y.S., Lin Y.E., Liu Y.-C., Hunag W.K., Shih H.Y., Wann S.R., Lee S.S., Tsai H.C., Li C.H., Chao H.L. (2008). Efficacy of point-of-entry copper–silver ionisation system in eradicating Legionella pneumophila in a tropical tertiary care hospital: Implications for hospitals contaminated with Legionella in both hot and cold water. J. Hosp. Infect..

[142] Thneibat A., Cochiran M.A., Gonzalez-Cabezas C., Moore B.K., Matis B.A., Lund M.R. (2008). Anticariogenic and antibacterial properties of a copper varnish using an in vitro microbial caries model. Oper. Dent..

[143] Mulligan A.M., Wilson M., Knowles J.C. (2003). The effect of increasing copper content in phosphate-based glasses on biofilms of Streptococcus sanguis. Biomaterials.

[144] Ditta I.B., Steele A., Liptrot C., Tobin J., Tyler H., Yates H.M., Sheel D.W., Foster H.A. (2008). Photocatalytic antimicrobial activity of thin surface films of TiO_2_, CuO and TiO_2_/CuO dual layers on Escherichia coli and bacteriophage T4. Appl. Microbiol. Biotechnol..

[145] Santo C.E., Taudte N., Nies D.H., Grass G. (2008). Contribution of copper ion resistance to survival of Escherichia coli on metallic copper surfaces. Appl. Environ. Microbiol..

[146] Abou Neel E.A., Ahmed I., Pratten J., Nazhat S.N., Knowles J.C. (2005). Characterisation of antibacterial copper releasing degradable phosphate glass fibres. Biomaterials.

[147] Guldiren D., Aydın S. (2017). Antimicrobial property of silver, silver-zinc and silver-copper incorporated soda lime glass prepared by ion exchange. Mater. Sci. Eng. C.

[148] Delgado K., Quijada R., Palma R., Palza H. (2011). Polypropylene with embedded copper metal or copper oxide nanoparticles as a novel plastic antimicrobial agent. Lett. Appl. Microbiol..

[149] Sudha V.P., Ganesan S., Pazhani G.P., Ramamurthy T., Nair G.B., Venkatasubramanian P. (2012). Storing drinking-water in copper pots kills contaminating diarrhoeagenic bacteria. J. Health Popul. Nutr..

[150] Sudha V.B.P., Singh K.O., Prasad S.R., Venkatasubramanian P. (2009). Killing of enteric bacteria in drinking water by a copper device for use in the home: Laboratory evidence. Trans. R. Soc. Trop. Med. Hyg..

[151] Sharan R., Chhibber S., Attri S., Reed R.H. (2010). Inactivation and sub-lethal injury of Escherichia coli in a copper water storage vessel: Effect of inorganic and organic constituents. Antonie Van Leeuwenhoek.

[152] Borkow G., Gabbay J. (2004). Putting copper into action: Copper- products with potent biocidal activities. FASEB J..

[153] Gabbay J., Borkow G., Mishal J., Magen E., Zatcoff R., Shemer-Avni Y. (2006). Copper oxide impregnated textiles with potent biocidal activities. J. Ind. Text..

[154] Ameh T., Sayes C.M. (2019). The potential exposure and hazards of copper nanoparticles: A review. Environ. Toxicol. Pharmacol..

[155] Gomes T., Pinheiro J.P., Cancio I., Pereira C.G., Cardoso C., Bebianno M.J. (2011). Effects of copper nanoparticles exposure in the musselmytilus galloprovincialis. Environ. Sci. Technol..

[156] Thounaojam T.C., Panda P., Mazumdar P., Kumar D., Sharma G., Saho L., Sanjib P. (2012). Excess copper induced oxidative stress and the response of antioxidants in rice. Plant Physiol. Biochem..

[157] Zhao L., Huang Y., Zhou H., Adeleye A., Wang H., Ortiz C., Mazer S., Keller A. (2016). GC-TOF-MS based metabolomics and ICP-MS based metallomics of cucumber (cucumis sativus) fruits reveal alteration of metabolites profile and biological pathway disruption induced by nano copper. Environ. Sci. Nano.

[158] Zhao L., Ortiz C., Adeleye A., Hu Q., Zhou H., Huang Y., Keller A. (2016). Metabolomics to detect response of lettuce (lactuca sativa) to Cu(OH)2 nanopesticides: Oxidative stress response and detoxification mechanism. Environ. Sci. Technol..

[159] Thomas T., Thomas K., Sadrieh N., Savage N., Adair P., Bronaugh R. (2006). Research strategies for safety evaluation of nanomaterials, part VII: Evaluating consumer exposure to nanoscale materials. Toxicol. Sci..

[160] Chibber S., Shanker R. (2017). Can CuO nanoparticles lead to epigenetic regulation of antioxydent enzyme system?. J. Appl. Toxicol..

[161] Sufian M.M., Khattak J.Z.K., Yousaf S., Rana M.S. (2017). Safety issues associatewith the use of nanoparticles in human body. Photodiag. Photodynam. Ther..

[162] Razavi M., Khandan A., Razavi M., Thakor A. (2017). Safety, regulatory issues, long-term biotoxicity, and the processing environment. Nanobiomaterials Science, Development and Evaluation.

[163] Madl A., Pinkerton K. (2009). Health effects of inhaled engineered and incidental nanoparticles. Crit. Rev. Toxicol..

[164] Lee I., Park S., Lim J., Shin I., Moon C., Kim S., Hero J., Kim J. (2016). Comparative toxicity and biodistribution of copper nanoparticles and cupric ions in rats. Int. J. Nanomedicine.

[165] Pra D., Franke S.I.R., Giulian R., Yoneama M.L., Dias J.F., Erdtmann B., Henriques J.A.P. (2008). Genotoxicity and mutagenicity of iron and copper in mice. Biometals.

[166] Bopp S.K., Abicht H.K., Knauer K. (2008). Copper-induced oxidative stress in rainbow trout gill cells. Aquat. Toxicol..

[167] Nawaz M., Manzl C., Lacher V., Krumschnabel G. (2006). Copperinduced stimulation of extracellular signal-regulated kinase in trout hepatocytes: The role of reactive oxygen species, Ca^2+^, and cell energetics and the impact of extracellular signalregulated kinase signaling on apoptosis and necrosis. Toxicol. Sci..

[168] Pandit A., Bhave S. (1996). Present interpretation of the role of copper in Indian childhood cirrhosis. Am. J. Clin. Nutr..

[169] Dietrich A.M., Glindemann D., Pizarro F., Gidi V., Olivares M., Araya M., Edwards M. (2004). Health and aesthetic impacts of copper corrosion on drinking water. Water Sci. Technol..

[170] Wang T., Wen X., Hu Y., Zhang X., Wang D., Yin S. (2019). Copper nanoparticles induced oxidation stress, cell apoptosis and immune response in the liver of juvenile Takifugu fasciatus. Fish Shellfish Immunol..

[171] Cheng T.C., Guida V.G., Butler M.S., Howland K.H. (2001). Use of copper compounds in shellfish depuration and disease control in mariculture. Incra Proj..

[172] Chandra S., Raizada S., Tyagi M., Gautam A. (2007). Synthesis, spectroscopic, and antimicrobial studies on bivalent nickel and copper complexes of bis (thiosemicrbazone). Bioinorg. Chem. Appl..

[173] Wilson N. (2018). Nanoparticles: Environmental problems or problem solvers?. Bioscience.

[174] Lewis A., Keevil C.W. (2004). Antibacterial Properties of Alloys and Its Alloys in HVAC&R Systems.

[175] Thurman R., Gerba C.A. (1989). Small sample of research and articles supporting the efficacy of silver as an antimicrobial agent follow: The molecular mechanisms of copper and silver ion disinfection of bacteria and viruses. CRC Crit. Rev. Environ. Control.

[176] Rosenberg M., Vija H., Kahru A., Keevil C.W., Ivask A. (2018). Rapid in situ assessment of Cu-ion mediated effects and antibacterial efficacy of copper surfaces. Sci. Rep..

[177] Warnes S.L., Caves V., Keevil C.W. (2012). Mechanism of copper surface toxicity in Escherichia coli O157: H7 and Salmonella involves immediate membrane depolarization followed by slower rate of DNA destruction which differs from that observed for Gram—Positive bacteria. Environ. Microbiol..

[178] Li M., Ma Z., Zhu Y., Yao M., Chu X., Wang X., Yang K., Yang M., Zhang Y., Mao C. (2016). Toward a molecular understanding of the antibacterial mechanism of copper—Bearing titanium alloys against staphylococcus aureus. Adv. Healthc. Mater..

[179] Vincent M., Hartemann P., Engels-Deutsch M. (2016). Antimicrobial applications of copper. Int. J. Hyg. Environ. Health.

[180] Santo C.E., Quaranta D., Grass G. (2012). Antimicrobial metallic copper surfaces kill Staphylococcus haemolyticus via membrane damage. Microbiologyopen.

[181] Langley S., Beveridge T.J. (1999). Effect of O-side-chain-lipopolysaccharide chemistry on metal binding. Appl. Environ. Microbiol..

[182] Fang L., Cai P., Chen W., Liang W., Hong Z., Huang Q. (2009). Impact of cell wall structure on the behavior of bacterial cells in the binding of copper and cadmium. Colloids Surf. A Physicochem. Eng. Asp..

[183] Chatterjee A.K., Chakraborty R., Basu T. (2014). Mechanism of antibacterial activity of copper nanoparticles. Nanotechnology.

[184] Slavin Y.N., Asnis J., Häfeli U.O., Bach H. (2017). Metal nanoparticles: Understanding the mechanisms behind antibacterial activity. J. Nanobiotechnol..

[185] Macomber L., Imlay J.A. (2009). The iron-sulfur clusters of dehydratases are primary intracellular targets of copper toxicity. Proc. Natl. Acad. Sci. USA.

[186] Lemire J.A., Harrison J.J., Turner R.J. (2013). Antimicrobial activity of metals: Mechanisms, molecular targets and applications. Nat. Rev. Microbiol..

[187] Peters K., Pazos M., Edoo Z., Hugonnet J., MArtorana A.M., Polissi A., VanNieuwenhze M.S., Arthur M., Vollmer W. (2018). Copper inhibits peptidoglycan LD-transpeptidases suppressing β-lactam resistance due to bypass of penicillin-binding proteins. Proc. Natl. Acad. Sci. USA.

[188] Warnes S.L., Keevil C.W. (2011). Mechanism of copper surface toxicity in vancomycin-resistant enterococci following wet or dry surface contact. Appl. Environ. Microbiol..

[189] Atiyeh B.S., Costagliola M., Hayek S.N., Dibo S.A. (2007). Effect of silver on burn wound infection control and healing: Review of the literature. Burns.

[190] Kuehl R., Brunetto P.S., Woisching A.-K., Varisco M., Rajacic Z., Vosbeck J., Terraciano L., Fromm K.M., Khanna N. (2016). Preventing implant-associated infections by silver coating. Antimicrob. Agent Chemother..

[191] Gibbard J. (1937). Public health aspects of the treatment of water and beverages with silver. Am. J. Public Health Nations Health.

[192] Luo J., Hein C., Mücklich F., Solioz M. (2017). Killing of bacteria by copper, cadmium, and silver surfaces reveals relevant physicochemical parameters. Biointerphases.

[193] Knobloch J.K., Tofem S., Kunz W., Schutze S., Riecke M., Solbach W., Wuske T. (2017). “Life-like” assessment of antimicrobial surfaces by a new touch transfer assay displays strong superiority of a copper alloy compared to silver containing surfaces. PLoS ONE.

[194] Xiu Z.M., Zhang Q.B., Puppala H.L., Colvin V.L., Alvarez P.J. (2012). Negligible particle-specific antibacterial activity of silver nanoparticles. Nano Lett..

[195] Hans M., Erbe A., Mathews S., Chen Y., Solioz M., Mücklich F. (2013). Role of copper oxides in contact-killing of bacteria. Langmuir.

[196] Champagne V.K., Helfritch D.J.A. (2013). Demonstration of the antimicrobial effectiveness of various copper surfaces. J. Biol. Eng..

[197] Scott R.D. (2009). The Direct Medical Cost of Healthcare-Associatedinfections in U.S Hospitals and the Benefits of Prevention.

